# Serial profiling of cell-free DNA and nucleosome histone modifications in cell cultures

**DOI:** 10.1038/s41598-021-88866-5

**Published:** 2021-05-04

**Authors:** Vida Ungerer, Abel J. Bronkhorst, Priscilla Van den Ackerveken, Marielle Herzog, Stefan Holdenrieder

**Affiliations:** 1 Institute for Laboratory Medicine, German Heart Centre, Technical University of Munich, Lazarettstraße 36, 80636 Munich, Germany; 2 Belgian Volition SRL, 22 Rue Phocas Lejeune, Parc Scientifique Crealys, 5032 Isnes, Belgium

**Keywords:** Biochemistry, Cancer, Cell biology, Molecular biology, Biomarkers, Molecular medicine, Oncology

## Abstract

Recent advances in basic research have unveiled several strategies for improving the sensitivity and specificity of cell-free DNA (cfDNA) based assays, which is a prerequisite for broadening its clinical use. Included among these strategies is leveraging knowledge of both the biogenesis and physico-chemical properties of cfDNA towards the identification of better disease-defining features and optimization of methods. While good progress has been made on this front, much of cfDNA biology remains uncharted. Here, we correlated serial measurements of cfDNA size, concentration and nucleosome histone modifications with various cellular parameters, including cell growth rate, viability, apoptosis, necrosis, and cell cycle phase in three different cell lines. Collectively, the picture emerged that temporal changes in cfDNA levels are rather irregular and not the result of constitutive release from live cells. Instead, changes in cfDNA levels correlated with intermittent cell death events, wherein apoptosis contributed more to cfDNA release in non-cancer cells and necrosis more in cancer cells. Interestingly, the presence of a ~ 3 kbp cfDNA population, which is often deemed to originate from accidental cell lysis or active release, was found to originate from necrosis. High-resolution analysis of this cfDNA population revealed an underlying DNA laddering pattern consisting of several oligo-nucleosomes, identical to those generated by apoptosis. This suggests that necrosis may contribute significantly to the pool of mono-nucleosomal cfDNA fragments that are generally interrogated for cancer mutational profiling. Furthermore, since active steps are often taken to exclude longer oligo-nucleosomes from clinical biospecimens and subsequent assays this raises the question of whether important pathological information is lost.

## Introduction

DNA is constantly being released from cells into the circulatory system as well as other bodily fluids^1,2^. In the last two decades there has been a considerable increase in the amount of effort and progress being made towards the optimal utilization of these cell-free DNA (cfDNA) molecules as minimally-invasive diagnostic and prognostic biomarkers for several pathologies and genetic aberrations, including many cancers^3,4^.

Elevated levels of total cfDNA does not appear to be a phenomenon unique to specific pathological states, as increased cfDNA concentrations have been demonstrated in patients with cancer^3,5^, sepsis^6^, cardiovascular disease^7^, autoimmune disease^8^, and stroke^9^, as well as in many benign or non-pathological conditions, such as pregnancy^10^, physical exercise^11–13^, and psychological stress^14,15^. Therefore, concentration-based measurements of total cfDNA alone are in many cases not deemed to be clinically meaningful. Instead, since cfDNA fragments conserve the genetic information from their cells of origin, the development of cfDNA assays has been driven forward rapidly through the implementation of ultrasensitive DNA mutational profiling technologies (i.e., NGS and ddPCR) and integrated bioinformatics approaches, which are now regarded the gold standard for the minimally-invasive detection of disease-specific genetic alterations^3,4^. While these methods have demonstrated unequivocal utility in some clinical settings and other scenarios, their diagnostic sensitivity and specificity are often challenged by biological limitations. For example, the presence of mutant cfDNA target molecules is often obscured by considerable background noise of wild-type cfDNA molecules, especially in early disease stages or other disease types where the source of mutant cfDNA is small. Moreover, the biological and physiological factors that modulate the cellular release of mutant vs non-mutant cfDNA molecules are poorly understood and underinvestigated (reviewed in^16^).

It is becoming increasingly recognized that the diagnostic sensitivity and specificity of mutation-based cfDNA assays can be enhanced by expanding the scope of tests to include the parallel characterization of various, potentially disease-specific, epigenetic features of cfDNA^17,18^, including (i) various classic epigenetic alterations such as histone modifications, histone variants, DNA methylation, and chromatin remodeling features^19–22^, (ii) fragment size; studies have shown that cfDNA fragment sizes differ between cancer patients (shorter fragments) and healthy controls (longer fragments)^23^, and have also been correlated with different mechanisms of release^3^, (iii) end-point fragmentation patterns and motifs^23–25^, and (iv) nucleosome density and spacing patterns^26–28^. An improved understanding of these physico-chemical features of cfDNA will not only elucidate optimal disease-defining biomarkers, but will enable biology-informed tailoring of extraction methods for optimal capturing of target molecules or exclusion of non-target molecules, and facilitate the optimization of preanalytical steps and customization of detection technologies and bioinformatics pipelines. In addition, a better understanding of the molecular origin and release mechanisms of cfDNA in different contexts will provide more insight into the factors that contribute to the extracellular presence of target vs non-target DNA and potentially open up a window for pharmacological intervention or the selection of patient conditions that either minimize the cellular release of background DNA or maximize the release of target DNA molecules into body fluids, thereby increasing chances for detection (reviewed in^29^).

Although progress is constantly being made in this regard, the molecular and cellular origin of cfDNA, the factors that modulate its release from cells, as well as its subsequent physico-chemical properties in different situations are still not fully characterized. Furthermore, the characterization of cfDNA is often complicated by the immense heterogeneity of both the in vivo setting and the aggregate cfDNA profile in a biospecimen. In an effort to circumnavigate these difficulties and gain a better understanding of cfDNA biology, a number of research groups including ourselves have implemented in vitro two-dimensional cell culture models, which offer a greater level of variable-control, to investigate the characteristics of cfDNA^30–38^. Unsurprisingly, this approach has already delivered some interesting insights. For example, a number of these cell culture studies have made quantitative and qualitative observations suggesting that a sub-population of cfDNA molecules may originate from live and dividing cells and exhibit characteristics distinct from those originating passively from accidental cell death or regulated cellular breakdown pathways such as apoptosis and necrosis^30,32,33,36,39^.

In this work we proceeded along these lines of investigation to probe deeper into the biology of cfDNA as it relates to physico-chemical properties, active vs passive release pathways, and differences between cell types. To do this we measured and compared the total cfDNA levels, fragment sizes, and epigenetic profiles of cell-free nucleosomes from three different cell lines (143B, PCS201010, and HMEC-1) under normal physiological conditions over time and correlated these values to the cell culture conditions including the cell counts, cell cycle phases, and levels of apoptosis and necrosis. The epigenetic alterations included histone variant H3.1, histone H3 acetylated at lysine 14 (H3K14ac), histone H3 tri-methylated at lysine 27 (H3K27me3), histone H4 acetylated at lysine 16 (H4K16ac), as well as histone variant H2AX phosphorylated at serine 139 (pH2AX).

## Materials and methods

### Cell culturing and processing of supernatant

The following cell lines were acquired from the American Type Culture Collection: Normal human primary dermal fibroblasts (ATCC PCS201010), human dermal microvascular endothelial cells (HMEC-1) (ATCC CRL-3243), and human bone cancer (osteosarcoma) cells (143B) (ATCC CRL-8303). The PCS201010 cells and 143B cells were grown in Dulbecco's modified Eagle's medium (Hyclone DMEM/high glucose) (Thermo Scientific, Waltham, MA, USA; cat# SH30243.01), with 4500 mg/L glucose, sodium pyruvate, and 4 mM L-glutamine. The HMEC-1 cells were grown in MCDB-131 culture medium (PAN Biotech, Aidenbach, Germany; cat# P04-80,057, lot# 3,390,618), with 1 µg/mL Hydrocortisone (Merck, Darmstadt, Germany; cat# H0888-1G, lot #SLBT5910), 20 mM L-Glutamine (Lonza, Basel, Switzerland; cat# 17-605E, lot# 8MB025), and 10 ng/mL EGF Recombinant Human Protein (Thermo Fisher Scientific, Waltham, MA, USA; cat# PHG0311, lot# 2,031,430). All the cell lines’ culture mediums were fortified with 10% fetal bovine serum (FBS) (PAN Biotech, Aidenbach, Germany; cat# P30-3302) and 1% penicillin/streptomycin (Lonza, Basel, Switzerland; cat# DE17- 602E, lot# 7MB159). The cells were incubated in controlled conditions at 37 °C in humidified atmosphere with 5% CO_2_. Cells were allowed to grow to confluency in two 175cm^2^ cell culture flasks (Thermo Fisher Scientific, Waltham, MA, USA; cat# 159,910). The cells were then washed with phosphate-buffered saline (PBS) (Sigma, St. Louis, USA; cat# SLBJ5110V) and trypsinized by incubating with 0.25% trypsin (Lonza, Basel, Switzerland; cat# 2MB258) for 10 min at 37 °C. The detached cells were then divided equally into 14 new 75 cm^2^ flasks (Thermo Fisher Scientific, Waltham, MA, USA; cat# 156,472, lot# 154,474), with each flask containing approximately 12 mL growth medium. The cells were then left to grow for exactly 12 h before the growth medium was replaced. From this time-point pairs of flasks were incubated for 4, 8, 12, 16, 20, 24, and 28 h for the 143B cells and for 4, 8, 12, 24, 36, 56, and 80 h, respectively, for both the PCS201010 and HMEC-1 cells. After each incubation period, the growth medium from each flask was collected in 15 mL nuclease-free conical tubes (CELLSTAR, Greiner Bio-One, Kremsmünster, Austria; cat# 1,882,714, lot# E16103T6), centrifuged at 1000 × g for 10 min, and transferred to new 15 mL tubes. These samples were aliquoted and stored at − 80 °C until further experiments were performed. The cells from the respective flasks were collected by trypsinization and used for various flow cytometric cell analyses including viability and apoptosis assays, and cell cycle analyses.

### Flow cytometry

A Guava Muse Cell Analyzer (Luminex Corp, Austin, TX, USA) benchtop flow cytometer was used for the laser-based detection of single cell fluorescence. This platform can evaluate up to 3 cellular parameters per event comprising 2 colors (detected in the red and/or yellow channels) and cell size (forward scatter). Gating strategies were used to distinguish the unstained populations from the fluorescently labeled cell populations, and a minimum of 5000 events were counted in most of the samples.

#### Cell count and viability measurement

The Muse Count & Viability Assay Kit (Luminex Corp, Austin, TX, USA; cat# MCH100102) was used for the quantitative analysis of cell viability and to determine the total amount of cells for each of the respective cell lines at the set time intervals described previously. The samples were prepared as recommended by the manufacturer, with slight modifications. Briefly, the cells were stained by mixing 100 µL of the cell suspension with 400 µL Muse Count & Viability Reagent, vortexed briefly, and incubated for 5 min at room temperature. The samples were briefly vortexed again immediately prior to measurement with the Guava Muse Cell Analyzer.

#### Caspase-3/7-based measurement of apoptosis and necrosis

The Muse Caspase-3/7 Kit (Luminex Corp, Austin, TX, USA; cat# MCH100108) was used for the simultaneous quantitative measurement of apoptotic status and cell death. This is achieved by utilizing a cell death dye for detection of cell membrane integrity and cell death together with a novel Muse Caspase-3/7 reagent, NucView 4, to check for Caspase-3/7 activity. The protocol was followed as stipulated in the manufacturer’s recommendations. Briefly, working solutions for both stock reagents were prepared as follows: The Muse Caspase-3/7 Stock solution was diluted 1:8 in PBS, and the Muse Caspase 7-AAD working solution was prepared by adding 148 μL of 1X Assay Buffer BA to 2 μL of 7-AAD Stock solution. Next, 200 µL of cell suspension was centrifuged at 300 × g for 5 min and the supernatant discarded. The cells were resuspended in 50 µL 1X Assay Buffer BA and 5 μL of the Muse Caspase-3/7 Reagent working solution was added to each sample. The samples were briefly vortexed at a medium speed and incubated in an incubator with 5% CO_2_ at 37 °C for 30 min. After the incubation period, 150 μL of the Muse Caspase 7-AAD working solution was added to each tube, mixed thoroughly by vortexing for 3 to 5 s, and incubated for 5 min in the dark at room temperature. After incubation, the samples were analyzed with the Guava Muse Cell Analyzer. The onboard software reports the results as both cell concentration and relative percentages of live, apoptotic, and necrotic cells in each sample.

#### Determination of cell cycle phase

The Muse Cell Cycle Assay Kit (Luminex Corp, Austin, TX, USA; cat# MCH100106) was used for cell cycle analyses. This assay relies on propidium iodide as a nuclear DNA intercalating stain and RNAse A to determine the fraction of total cells in each cell cycle phase (G0/G1, S, and G2/M). The cells are prepared and stained according to the user manual, with minor modifications. Briefly, 1200 μL of cell suspension was added to a 2 mL microcentrifuge tube, centrifuged at 300 × g for 5 min, and washed once with 1500 μL of 1X PBS. The cells were resuspended by slowly adding 1500 μL of ice-cold 70% (v/v) ethanol to the washed pellet, and vortexed briefly. The cell suspensions were incubated at least overnight at –20 °C before analysis. Immediately prior to analysis, 200 μL of Muse Cell Cycle Reagent was added to each sample, which was then incubated at room temperature, protected from light for 30 min. After the incubation time, samples were analyzed for DNA fragmentation with Guava Muse Cell Analyzer. The instrument software expressed the results as a percentage of the total cells in each phase of the cell cycle, G0/G1, S, or G2/M, as well as the mean and the percentage of the coefficient of variation (%CV) values for the fluorescence intensity.

### Cell-free DNA isolation

The NucleoSpin Gel and PCR Clean-up kit (Macherey–Nagel, Düren, Germany; cat# 740,609,250, lot# 1801/007) was used for the isolation of cfDNA from the cell culture supernatants, conforming to the manufacturer's PCR clean-up instructions, with minor modifications. In short, the samples were thawed at 37 °C in a water bath, vortexed briefly and spun to collect droplets from the caps. For each flask (biological replicate), duplicate extractions were done on 1.7 mL culture supernatant. Samples were mixed with 3.4 mL Buffer NTB, in a sample-to-buffer ratio of 1:2, and vortexed on medium speed for 5 s. For extraction, the spin columns were filled with 600 μL of culture media and centrifuged at 11,000 × g for 1 min at room temperature. This was repeated until the whole volume of each sample was passed through the column. The columns were washed with a wash buffer twice and cfDNA eluted into 20 μL elution buffer.

### Quantification of nucleosomes containing the H3.1 variant and specific modifications

Five nucleosome structures were measured using Nu.Q Immunoassays (Belgian Volition SRL, Namur, Belgium) according to the manufacturer’s instructions. These sandwich immunoassays, which are based on magnetic beads and chemiluminescence technology, was performed on the IDS-i10 automated immunoanalyzer system (Immunodiagnostic Systems Ltd (IDS), UK). Briefly, 50 µL of cell culture supernatant aliquots were incubated with acridinium ester labeled anti-nucleosome antibody. After this incubation step the magnetic particle beads, coated with the corresponding monoclonal anti-histone modification capture antibody (i.e. anti-histone H3.1, anti-histone H3K27me3, anti-histone H3K14ac, anti-histone H4K16ac, or anti-histone pH2AX, respectively), were added. Following this second incubation, sandwich complexes comprising the anti-nucleosome antibody, nucleosome, and magnetic particle were separated from the unbound nucleosomes using a magnet. After a wash step, trigger reagents were added, and the light emitted by the acridinium ester was measured and expressed in relative light unit (RLU) by the system luminometer. All samples were analyzed in duplicate. If the %CV between the RLU of the duplicate measurements was above 20%, the sample was repeated. Histone modification concentrations were determined using a four-parameter logistic regression of a reference standard curve. Results are provided in Unit (U)/mL, as there is no international standard for circulating nucleosomes available yet. As each individual assay has its own standard, direct comparisons of the amounts measured are not possible between the assays.

### Cell-free DNA quantification

#### Qubit assay

The Qubit dsDNA HS Assay Kit (Invitrogen, Life Technologies, Carlsbad, CA, USA; cat# Q32851, lot# 1,724,782) was used for the quantification of cfDNA on the Qubit 3.0 Fluorometer (Invitrogen, Life Technologies, Carlsbad, CA, USA). A standard curve was prepared, as per the manufacturer’s guidelines, using the zero and 10 ng/μL Qubit kit standards. For all cfDNA samples 197 µL Qubit working solution was mixed with 3 µL of cfDNA, briefly vortexed, and incubated at room temperature for 2 min before measurement.

#### Real-time PCR

A real-time quantitative (qPCR) assay was used for the measurement of PCR amplification of cfDNA using the β-globin gene. Assays were performed with a LightCycler 480 Instrument II (Roche, Basel, Switzerland) in a 96-well plate setup. The reaction mixtures consisted of 1 μL DNA and 24 μL master mix, which comprised 12.5 μL TaqMan Universal MasterMix (Applied Biosystems, Foster City, CA, USA; cat# 4,304,437, lot# 1,805,142), 9.1 μL RT-PCR grade H_2_O (Invitrogen, Carlsbad, CA, USA; cat# AM9935, lot# 1,804,029), 0.4 μL of 10 μM dual fluorescent probe 5′- (FAM)AAG GTG AAC GTG GAT GAA GTT GGT GG(TAMRA)-3′, and 1 μL of 10 μM forward and reverse primers, respectively. The primers used were: F1, 5′-GTG CAC CTG ACT CCT GAG GAG A-3′, and R1, 5′-CCT TGA TAC CAA CCT GCC CAG-3′. The primers and probe were manufactured by TIB MOLBIOL (Berlin, Germany). Sequence data of the β-globin gene is accessible from GenBank (accession number: U01317). The PCR conditions were the following: 95 °C for 10 min, followed by 45 cycles of a 15 s denaturation step at 95 °C, a 1 min annealing step at 60 °C, and a 30 s extension step at 72 °C. A five-point standard curve, consisting of five different genomic DNA dilutions (Applied Biosystems, Foster City, CA, USA; cat# 4,312,660, lot# 360,486) (25, 250, 1000, 2500, and 10,000 pg/μL respectively) was used for the absolute quantification of the target gene. The standard curve was prepared in triplicate and each biological replicate was quantified in duplicate. Only assays where the R^2^ values for the standard curve were above 0.99 were included.

### Cell-free DNA size analysis

The Agilent 2100 Bioanalyzer (Agilent Technologies Inc., Santa Clara, CA, USA) equipped with Expert 2100 software was used for the size analysis of cfDNA by means of capillary electrophoresis (CE). The High Sensitivity DNA microchip (Agilent Technologies, cat# 5067–4627, lot# WG23BK50) and High Sensitivity DNA kit (Agilent Technologies Inc., Santa Clara, CA, USA, cat# 5067–4627, lot# 1834) were used for size separation. The assay was performed according to the instructions stated in the user manual. Additional size analyses were performed using the dsDNA 930 Reagent kit (Agilent Technologies Inc., Santa Clara, CA, USA) on the Fragment Analyzer system (Agilent Technologies Inc., Santa Clara, CA, USA). These assays include a ladder and two DNA markers, which is used by the software to automatically calculate the size of the cfDNA fragments, based on the relative sizes and retention times of each of the virtual bands generated by the software. In order to assess the relative contribution of differently sized cfDNA populations toward the total population, as well as determine average sizes, the percentage and average size of cfDNA fragments that lie within five selected fragment size ranges were calculated using the size-gating function of the Expert 2100 software. The 5 selected size ranges were (i) 50–250 bp (ii) 250–450 bp, (iii) 450–650 bp, and (iv) 650–10,000 bp.

### Statistics and data representation

All statistics and data visualization were performed using GraphPad Prism version 5.00 for Windows, GraphPad Software, San Diego, California USA, www.graphpad.com. Grubbs’ test was used to identify outliers. The Pearson correlation coefficient (r-value) was used to determine linear correlations between different variables. Only p-values < 0.05 were considered to be statistically significant.

## Results and discussion

The goal of this work was to gain an improved understanding of the molecular origin and characteristics of cfDNA in cell cultures. To do this we performed a longitudinal assessment of cfDNA in three different cell lines, including (i) human bone osteosarcoma (143B), (ii) primary dermal fibroblasts (PCS201010), and (iii) human dermal microvascular endothelial cells (HMEC-1). For each of these cell lines, both the cells and cell culture supernatant were collected at specific time-points after increasing periods of incubation. For each time-point cells were counted, assessed for viability, apoptosis and necrosis, and cell cycle phase. At the corresponding time-points the supernatant was assessed for total cfDNA levels, cfDNA fragment size distribution, and levels of cell-free nucleosomes containing the H3.1 variant as well as specific post-translational histone modifications (PTMs), including H3K27me3, H3K14ac, H4K16ac and pH2AX. In the following section we present the absolute values that were measured and discuss correlations between these measurements.

### Total cell counts and cell cycle phase

**Figure 1:**
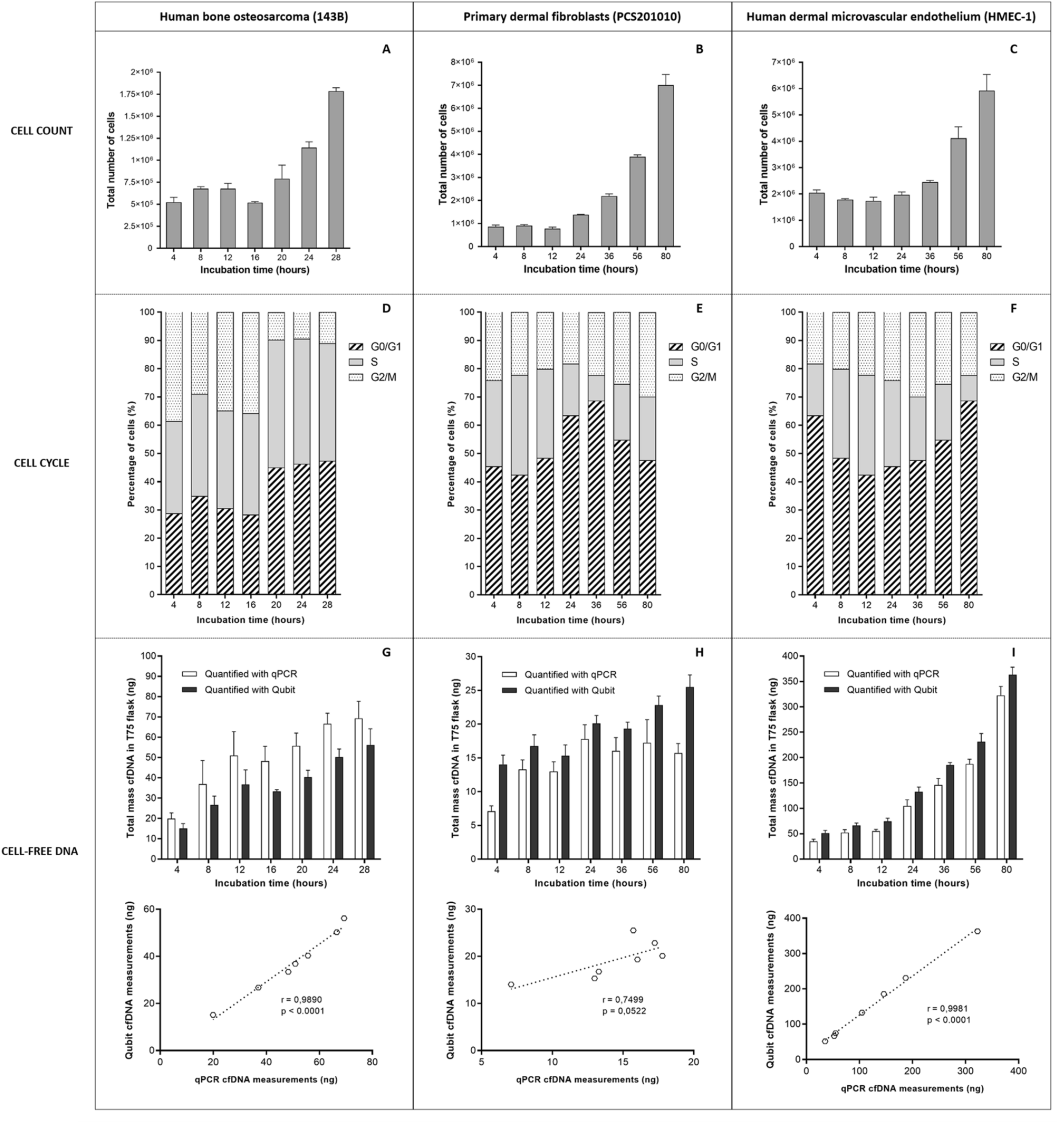
Longitudinal measurements of cell number, cell cycle and total cell-free DNA in three cell lines. A cell count and viability assay was used to determine the total number of cells present in a T-75 culture flask after different incubation times for (**A**) human bone osteosarcoma (143B), (**B**) primary dermal fibroblasts (PCS201010), and (**C**) human dermal microvascular endothelial cells (HMEC-1). Changes in the phase of cell cycle over increasing incubation times were measured in (**D**) 143B, (**E**) PCS201010, and (**F**) HMEC-1 cells using a cell cycle assay kit. The relative number of cells in the G0/G1, S, or G2/M phases, respectively, at each incubation time-point is expressed as a percentage of the whole. CfDNA was collected from (**G**) 143B, (**H**) PCS201010, and (**I**) HMEC-1 cell culture supernatant after different incubation times and quantified with a β-globin based PCR assay (white bars) and Qubit dsDNA HS Assay (black bars), respectively. The values are expressed as the total amount of cfDNA (ng) present in a T-75 culture flask. The statistical correlation between qPCR and Qubit measurements for each cell line is shown below the respective cfDNA quantification graphs. Each time-point bar represents the mean values of two biological replicates (measurements made from two separate cell culture flasks). Error bars indicate standard deviation. R-values close to 1 indicate perfect correlation, while R-values close to zero indicate no correlation. P-values < 0.05 indicate statistical significance.

As measured by the Guava Muse Cell Analyzer using the Muse Count & Viability Kit, the number of cells remained either stable or showed a slow growth rate in early incubation periods and increased significantly for each cell line after a specific period of incubation: between 16–24 h for 143B cells, 12–24 h for PCS201010 cells, and 24–36 h for HMEC-1 cells (Fig. 1A–C). The time-points at which the process of cell division was initiated for each cell line is reflected by corresponding shifts in their cell cycle phases, wherein populations of cells can be seen to shift out of the S or G2/M phases and into the G0/G1 phase of the cell cycle over increasing time periods (Fig. 1D–F). It should be noted here that all three cell lines were cultured under normal conditions. As such a large portion of cells are naturally not synchronized in their cell cycles and divide irregularly throughout the entire incubation period, which results in a broad cell cycle distribution. Therefore, as it is not expected that the majority of cells will be in the same cell cycle phase at the same time or transition simultaneously, a perfect correlation between cell number and cell cycle phase is not expected.

### Total cfDNA, H3.1 and PTM levels

As determined by qPCR and Qubit DNA quantification assays, respectively, total cfDNA levels for each cell line increased incrementally throughout the entire incubation period, reaching maximum levels at the end of the experiment (Fig. 1G–I). Furthermore, qPCR and Qubit measurements correlated significantly for 143B cells (r = 0.99) (Fig. 1G) and HMEC-1 cells (r = 0.99) (Fig. 1I) but did not correlate strongly for PCS201010 cells (r = 0.75) (Fig. 1H), in which case qPCR cfDNA levels plateaued after 24 h of incubation, while Qubit cfDNA levels continued to rise. The reason for this is unclear, but a previous study has also demonstrated significant differences in the representation of house-keeping gene sequences, including β-globin, in the total cfDNA population in the same cell line and between cell lines^40^. Similarly, total cfDNA levels measured by qPCR is significantly greater than the total levels of cfDNA measured by the Qubit assay in 143B cells at each time-point (Fig. 1 G), while the opposite is true for HMEC-1 and PCS201010 cells. The reason why the β-globin gene sequence is overrepresented in the total cfDNA population in 143B cells in comparison with HMEC-1 cells and PCS201010 cells is also unclear but may relate to differences in copy-number or expression of β-globin. Studies have, for example, demonstrated overexpression of β-globin in cancer cells that exhibit a higher level of aggression and metastatic potential^41,42^.

**Figure 2:**
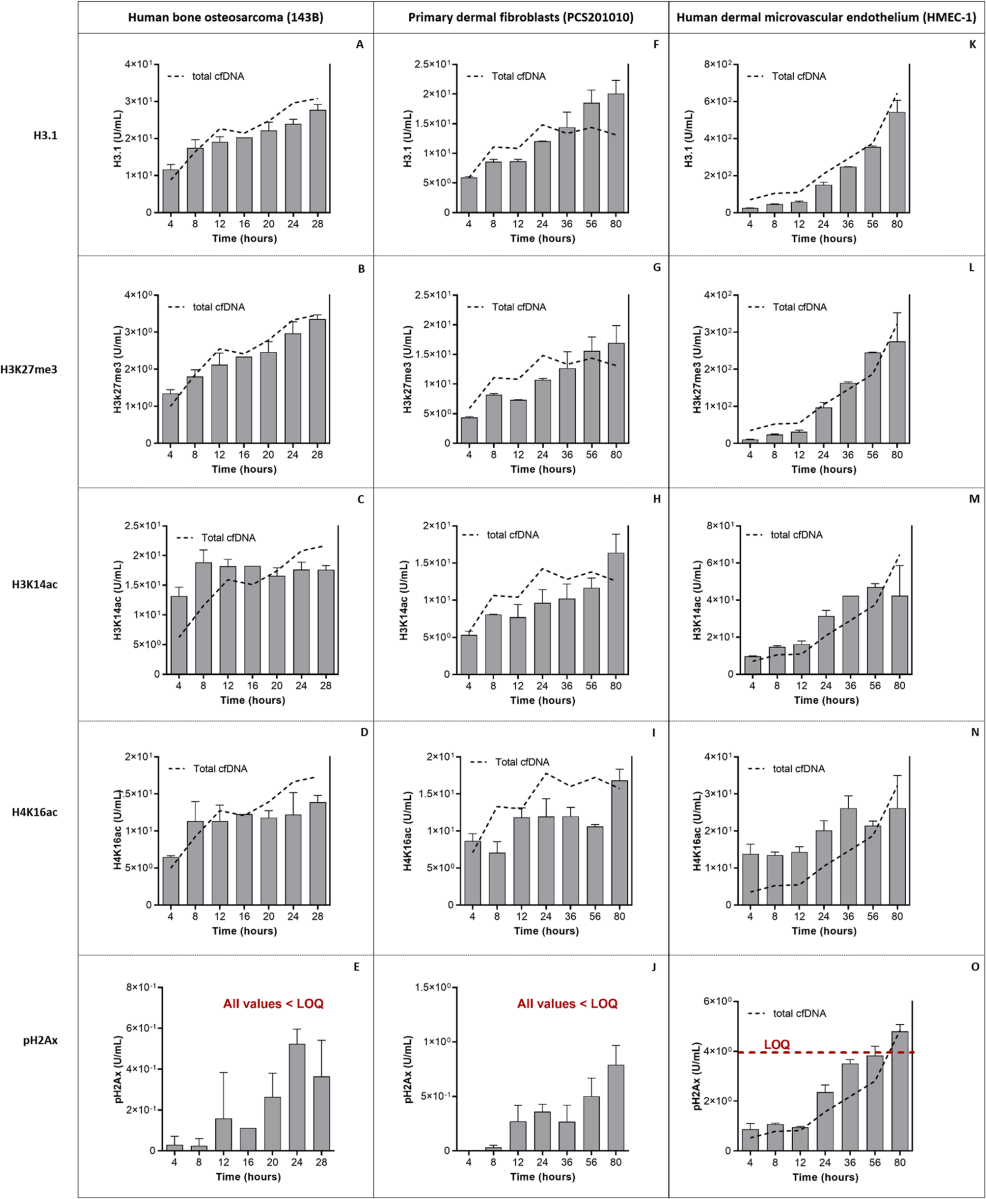
Longitudinal measurements of nucleosomal H3.1 and histone PTMs in three cell lines. Using chemiluminescence based Nu.Q Immunoassays, total levels of H3.1, H3K27me3, H3K14ac, H4K16ac and pH2AX, respectively, were measured directly from 50 µL of cell culture supernatant collected at different time-points after increasing periods of incubation from **(A-E)** human bone osteosarcoma (143B) cells, **(F-J)** primary dermal fibroblasts (PCS201010), and **(K–O)** human dermal microvascular endothelial cells (HMEC-1). For all cell lines, each time-point bar represents the mean of measurements from two biological replicates (i.e., parallel characterization of two separate cell culture flasks), except for the 16 h time-point in 143B cells, for which data from one of the flasks was compromised due to experimental error and omitted. Error bars indicate standard deviation. As indicated in red text in Figs E, J and O, all measured pH2AX values were under the limit of quantification (LOQ) of the assay, except for the 80 h time-point in HMEC-1 cells. The dotted black lines overlayed on each graph illustrate the corresponding pattern of total cfDNA levels, as measured by a qPCR assay (data from Fig. 1G–I).

As determined by the Nu.Q Immunoassays, levels of cell-free nucleosomes containing H3.1 and the different PTMs increased throughout the entire incubation period in each cell line, also reaching maximum levels at the end of the experiment (Fig. 2), with the exception of H3K14ac in 143B cells which maintained constant levels throughout the experiment (Fig. 2 C). Levels of pH2AX were below the limit of quantification in all cell lines and were thus excluded from further analyses. As shown in Fig. 2 and Table 1, there was a strong correlation between total cfDNA levels and H3.1 and the different PTMs. This indicates that the majority of cfDNA fragments present in the cell culture supernatant is not “naked”, so to speak, but exists in nucleosomal structures that harbor the H3.1 variant as well as the H3K27me3, H3K14ac, and H4K16ac modifications. Thus, for the remainder of the paper cfDNA and cell-free nucleosomes may be considered one unit and interchangeable terms. Two interesting observations here, however, is that (i) total cfDNA levels do not correlate with H3K14ac in 143B cells, and not with H3K16ac in PCS201010 cells (when measured with qPCR), and (ii) in line with the latter observation, total cfDNA measurements as determined by the Qubit assay in PCS201010 cells showed a much stronger correlation with H3.1, H3K27me3, H3K14ac, and H4K16ac levels than qPCR cfDNA measurements.

**Table 1:** Correlation between changes in total cfDNA levels vs. total cell-free nucleosomal H3.1, H3K27me3, H3K14ac, and H4K16ac levels, respectively, over increasing incubation periods in three cell lines.

Cell line	Total cfDNA	Pearson's correlation coefficient (r)			
		H3.1	H3K27me3	H3K14ac	H4K16ac
143B	qPCR	0.97	0.97	0.57	0.89
	Qubit	0.98	0.98	0.50	0.86
PCS201010	qPCR	0.74	0.79	0.67	0.47
	Qubit	0.97	0.97	0.96	0.72
HMEC-1	qPCR	0.99	0.95	0.82	0.84
	Qubit	1.00	0.96	0.85	0.86

### Cell growth and integrity vs total cfDNA and nucleosome H3.1 and PTM levels

The total number of cells (Fig. 1A–C), total cfDNA (Fig. 1G–I), as well as total cell-free nucleosomal H3.1 and histone PTM levels (barring H3K14ac in 143B cells) (Fig. 2), respectively, showed a trend of incremental increase over increasing incubation periods for all cell lines. Accordingly, total cell numbers vs total cfDNA and total cell-free nucleosomal H3.1 and histone PTM levels, respectively, showed moderate to strong correlations for all cell lines (Supplementary Figures S1 and S2). However, based on both qPCR and Qubit measurements, changes in total cfDNA levels between incubation periods did not match changes in the absolute number of live cells in the same period in any of the cell lines (Table 2). In 143B cells cfDNA levels increase significantly between 0–16 h of incubation, whereas the number of live cells remain relatively stable. In contrast, between 16–28 h of incubation, the significant increase in live cells is not matched by an equally prominent increase in total cfDNA levels. For example, between 16–20 h, 20–24 h, and 24–28 h of incubation, total cfDNA levels increased by 15%, 20%, and 4% (qPCR assay), respectively, while the total number of live cells increased by 51%, 44%, and 58%, respectively. This lack of correlation was also seen in PCS201010 and HMEC-1 cells, which indicated that DNA is not constitutively released into the cell culture supernatant from live and dividing cells by any of the examined cell lines (Table 2). Given the lack of correlation between total cfDNA and live cells, we next assessed whether cfDNA levels instead correlated with changes in the levels of apoptotic or necrotic cells.

**Table 2:** Percentage increase/decrease in total cfDNA levels over each incubation period vs the percentage increase/decrease in the total number of live cells over the corresponding periods.

Human bone osteosarcoma (143B) cells							
Time-segment	Change in total live cells (%)	Change in total cfDNA (%)					
	Count and viability assay	qPCR	Pearson's r	p-value	Qubit	Pearson's r	p-value
4 to 8 h	33.18	85.52	0.11	0.84	76.70	0.2509	0.63
8 to 12 h	−0.65	37.79			37.85		
12 to 16 h	−23.01	−5.28			−9.28		
16 to 20 h	51.09	15.41			20.76		
20 to 24 h	43.61	19.51			24.61		
24 to 28 h	57.89	4.16			11.75		

Primary dermal fibroblasts (PCS201010)							
Time-segment	Change in total live cells (%)	Change in total cfDNA (%)					
	Count and viability assay	qPCR	Pearson's r	p-value	Qubit	Pearson's r	p-value
4 to 8 h	7.08	87.36	−0.31	0.55	19.26	0.51	0.30
8 to 12 h	−15.57	−2.24			−8.57		
12 to 24 h	79.11	36.97			31.24		
24 to 36 h	58.49	−9.96			−3.85		
36 to 56 h	76.02	7.67			18.20		
56 to 80 h	82.80	−8.82			11.68		

Human dermal microvascular endothelial cells (HMEC-1)							
Time-segment	Change in total live cells (%)	Change in total cfDNA (%)					
	Count and viability assay	qPCR	Pearson's r	p-value	Qubit	Pearson's r	p-value
4 to 8 h	−14.02	48.74	0.08	0.88	29.66	0.13	0.80
8 to 12 h	−2.23	4.98			12.04		
12 to 24 h	14.20	90.66			77.59		
24 to 36 h	24.61	39.23			39.86		
36 to 56 h	65.80	28.36			24.61		
56 to 80 h	44.76	72.24			57.28		

### Apoptosis vs necrosis in the generation of cfDNA

**Figure 3:**
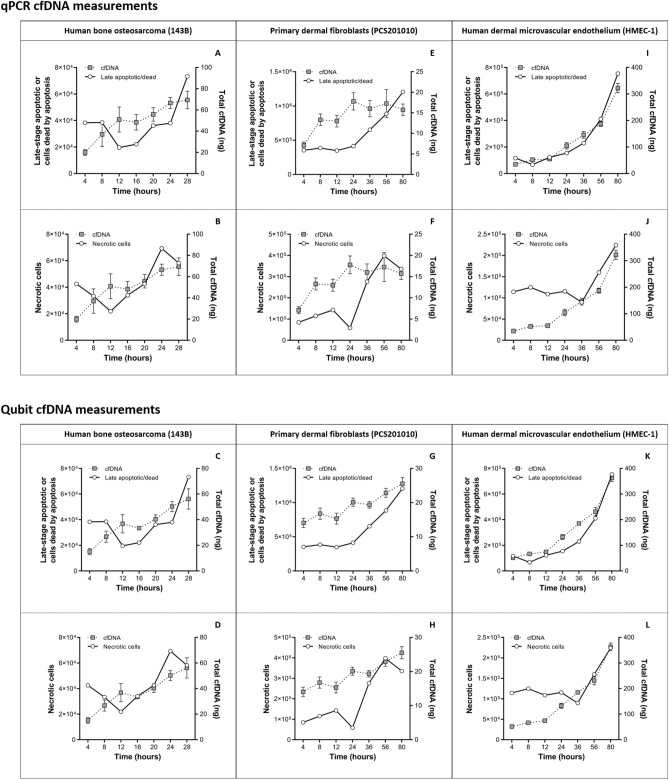
Total cfDNA levels vs total number of apoptotic and necrotic cells. Changes in total cfDNA levels as measured by both the β-globin-based qPCR (**top**) and Qubit HS DNA (**bottom**) assays over increasing incubation periods was plotted against corresponding changes in the total numbers of apoptotic and necrotic cells, respectively, as determined by a caspase-3/7 assay. **(A-D)** Human bone osteosarcoma (143B), **(E–H)** primary dermal fibroblasts (PCS201010), and **(I-L)** human dermal microvascular endothelial (HMEC-1) cell lines were investigated to observe the time-dependent changes in absolute cfDNA levels and corresponding changes in the total number of late-stage apoptotic cells or cells already dead by apoptosis (**A**, **E**, **I**, **C**, **G**, **K**), and necrotic cells (**B**, **F**, **J**, **D**, **H**, **L**). cfDNA values represent the total mass (ng) of cfDNA in the cell culture supernatant after each incubation period. Linear regression analyses for total cfDNA vs total apoptotic and necrotic cells are summarized in Supplementary Table S2.

The number of both apoptotic and necrotic cells increased over time and was matched by increases in total cfDNA levels in all cell lines (Fig. 3). This indicated that both apoptosis and necrosis is involved in the generation of cfDNA in all cell lines. Therefore, we next investigated the relative contribution of apoptotic and necrotic processes towards the total cfDNA pool. The number of apoptotic and necrotic cells showed different dynamics over increasing incubation periods (Fig. 3). Linear regression analysis over the entire incubation period showed a moderate correlation for 143B cells (r = 0.63), and strong positive correlations for PCS201010 (r = 0.87) and HMEC-1 cells (r = 0.91) (Supplementary Table S1). However, this statistical model was not appropriate for describing the clear fluctuations in the proportion of apoptotic and necrotic cells at specific time points during the time-course experiment, as seen in Fig. 3. For example, changes in the number of apoptotic and necrotic cells in 143B cells showed a moderate correlation between 4–16 h of incubation (r = 0.73), but a weak correlation between 16–28 h of incubation (r = 0.52). In PCS201010 cells, changes in apoptotic and necrotic cells showed a moderate negative correlation between 4–24 h of incubation (r = −0.67), but a strong positive correlation between 24–80 h of incubation (r = 0.79). Similarly, in HMEC-1 cells changes in apoptotic and necrotic cells showed a strong negative correlation between 4–36 h of incubation (r = −0.93), but a near-perfect positive correlation between 36–80 h of incubation (r = 0.98) (Supplementary Table S1). As will be discussed next, some of these changes in the relative proportion of apoptotic vs necrotic cells were reflected by changes in both total cfDNA levels and the relative proportion of differently sized cfDNA populations in all cell lines.

### Human dermal microvascular endothelial cells (HMEC-1)

Changes in total cfDNA levels, as determined by qPCR, showed a greater correlation with changes in the number of apoptotic cells (r = 0.98) than with necrotic cells (r = 0.84) over the entire 80 h incubation period (Fig. 3 I–L and Supplementary Table S2). However, up to 56 h total cfDNA levels showed no correlation with necrotic cells (r = 0.38), but still correlated significantly with apoptotic cells (r = 0.92) (Supplementary Table S2). These differences were reflected by changes in the levels of specific cfDNA size populations. Over the entire 80 h incubation period, changes in total cfDNA levels showed a greater correlation with changes in the levels of 50–250 bp (r = 0.97), 250–450 bp (r = 0.99) and 450–650 bp (r = 0.96) cfDNA fragments than with cfDNA fragments ranging between 650–10,000 bp (r = 0.88) (Supplementary Table S3. However, changes in the levels of 650–10,000 bp cfDNA fragments did not correlate with total cfDNA up until 56 h of incubation (r = 0.52), while 50–250 bp (r = 0.99), 250–450 bp (r = 0.98), and 450–650 bp (r = 0.9) cfDNA fragments correlated significantly with total cfDNA (Supplementary Table S3). The same results were obtained for total cfDNA measured by the Qubit assay (Supplementary Table S3).

Similar to changes in total cfDNA levels, changes in 50–250 bp (r = 0.93), 250–450 bp (r = 0.94) 450–650 bp (r = 0.95), and 650–10,000 bp (r = 0.91) cfDNA fragments showed a greater correlation with changes in the number of apoptotic cells than necrotic cells over the entire 80 h incubation period (Supplementary Table S4). However, up to 56 h of incubation changes in 50–250 bp (r = 0.43), 250–450 bp (r = 0.26) and 450–650 bp (r = 0.2) cfDNA fragments did not correlate with necrotic cells but did correlate with apoptotic cells (Supplementary Table S4). In contrast, changes in the level of 650–10,000 bp cfDNA fragments between 0–56 h of incubation did not correlate with changes in the number of apoptotic or necrotic cells. The reason for this is likely that larger cfDNA fragments did not increase significantly over the entire incubation period, except between 56–80 h of incubation when it increased significantly (Table 3). Similarly, the total number of necrotic cells did not increase significantly over the entire incubation period, except after 36-80 h of incubation (Fig. 3J,L). Therefore, it is likely that the first large population of long cfDNA fragments were produced in the first 4 h of incubation, followed by minor increase up until 56 h of incubation. The second large population of long cfDNA fragments were then produced between 56–80 h incubation after some delay between the rapid increase of new necrotic cells and eventual disintegration and release of their genetic material. This relationship is in contrast to the incremental increase of short cfDNA fragments (Table 3) that are matched by incremental increases in apoptotic cells (Fig. 3I,K). As will be discussed later, changes in the levels of longer cfDNA fragments over time may have been affected by degradation.

**Table 3:** Details of cfDNA size determinations. including the concentration of differently sized cfDNA populations and their relative percentage contributions. and the average fragment sizes over time.

Cell line	Parameter	Time point	Size range			
			50–250 bp	250–450 bp	450–650 bp	650–10.000 bp
Human bone osteosarcoma (143B)	Average size (bp)	4 h	138.26	341.13	553.55	3673.28
		8 h	120.60	383.39	531.31	3401.20
		12 h	198.96	358.61	548.29	2708.10
		16 h	158.35	356.77	552.52	3441.18
		20 h	167.13	362.85	550.13	2909.26
		24 h	182.14	355.53	543.87	2975.03
		28 h	181.19	351.04	541.61	3063.85
	Concentration (pg/µl) (% of total)	4 h	87.00 (23.8%)	27.10 (9.5%)	16.00 (6.4%)	104.32 (50.7%)
		8 h	10.03 (3.6%)	19.92 (9.7%)	14.50 (7.6%)	111.52 (72.3%)
		12 h	30.34 (4.3%)	89.34 (15.2%)	84.38 (15.9%)	273.26 (62.0%)
		16 h	62.12 (5.8%)	51.74 (6.1%)	52.27 (6.9%)	468.32 (76.5%)
		20 h	51.42 (7.4%)	65.00 (11.8%)	71.30 (14.3%)	251.65 (60.7%)
		24 h	80.18 (9.8%)	122.43 (18.3%)	101.46 (16.9%)	256.14 (51.0%)
		28 h	98.16 (12.8%)	135.22 (21.6%)	95.35 (17.0%)	207.37 (44.2%)
Primary dermal fibroblasts (PCS201010)	Average size (bp)	4 h	130.05	347.71	539.44	4051.61
		8 h	119.72	368.47	547.12	3641.26
		12 h	146.91	347.03	550.55	3314.38
		24 h	165.17	348.09	543.99	2757.97
		36 h	170.22	344.58	537.93	3140.73
		56 h	161.72	336.04	534.49	3617.12
		80 h	158.41	334.16	537.96	3327.41
	Concentration (pg/µl) (% of total)	4 h	120.65 (40.0%)	18.52 (8.0%)	4.87 (2.4%)	61.13 (36.8%)
		8 h	56.00 (16.8%)	13.99 (5.7%)	13.01 (5.8%)	116.16 (63.8%)
		12 h	93.28 (21.8%)	34.58 (10.3%)	28.63 (9.6%)	125.40 (50.8%)
		24 h	106.25 (19.3%)	82.21 (18.6%)	56.58 (14.3%)	138.04 (41.9%)
		36 h	120.83 (22.6%)	118.03 (27.2%)	62.61 (16.2%)	95.89 (29.6%)
		56 h	113.30 (31.0%)	69.66 (23.6%)	28.45 (10.9%)	56.13 (26.0%)
		80 h	219.14 (32.8%)	112.21 (20.8%)	44.40 (9.4%)	108.71 (27.8%)
Human dermal microvascular endothelial (HMEC-1)	Average size (bp)	4 h	183.65	355.13	540.79	2758.07
		8 h	177.92	348.57	536.88	2857.76
		12 h	171.27	351.63	535.63	3040.59
		24 h	165.71	339.61	528.42	2779.56
		36 h	162.43	338.37	529.08	2813.12
		56 h	157.46	335.42	528.85	2830.32
		80 h	158.79	335.84	531.37	2931.37
	Concentration (pg/µl)	4 h	128.87 (23.8%)	86.24 (19.5%)	54.33 (13.7%)	137.08 (41.7%)
		8 h	304.04 (33.6%)	148.23 (20.1%)	74.62 (11.3%)	181.65 (33.4%)
		12 h	318.92 (37.7%)	115.90 (17.0%)	62.95 (10.3%)	160.31 (31.9%)
		24 h	1053.96 (61.5%)	232.14 (16.7%)	72.87 (5.9%)	133.75 (13.1%)
		36 h	1780.02 (65.4%)	337.72 (15.4%)	94.49 (4.8%)	199.83 (12.6%)
		56 h	2612.28 (72. 7%)	360.80 (12.5%)	92.88 (3.6%)	182.36 (8.7%)
		80 h	3370.33 (65.2%)	569.52 (13.7%)	167.93 (4.6%)	427.10 (14.3%)

**Figure 4:**
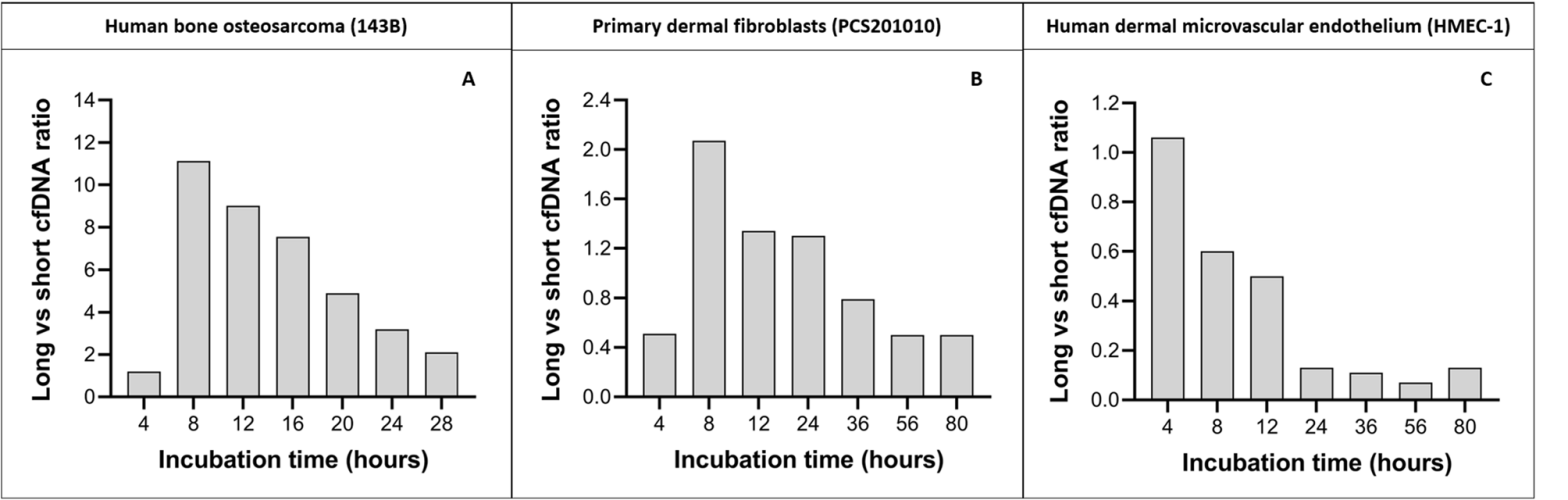
Ratio of long to short cfDNA fragments. Cell-free DNA (cfDNA) size profiles for each time-point were generated using an Agilent Bioanalyzer and High Sensitivity DNA kit. The size profiles were gated into two groups corresponding to the total contribution (pg/µl) of both short (50–250 bp) and long (650–10,000 bp) cfDNA populations using the size-gating function of the onboard Expert 2100 software. To illustrate the relationship between these populations we plotted the ratio of long to short cfDNA fragments over time for **(A)** Human bone osteosarcoma (143B), **(B)** primary dermal fibroblasts (PCS201010), and **(C)** human dermal microvascular endothelial (HMEC-1) cell lines.

**Figure 5:**
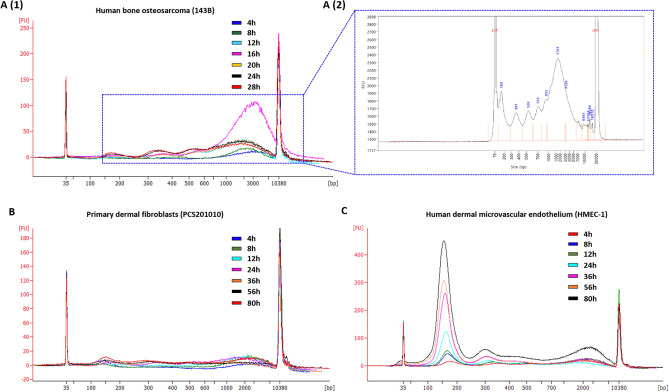
Cell-free DNA was isolated directly from cell culture supernatants and subject to size analysis. Electropherograms, generated with an Agilent Bioanalyzer, illustrates the cell-free DNA (cfDNA) size profiles at each time-point in cell culture supernatants from (**A**) human bone osteosarcoma (143B), (**B**) primary dermal fibroblast (PCS201010), and (**C**) human dermal microvascular endothelial (HMEC-1) cell lines. Electropherogram **A (2)** shows a 143B DNA size profile obtained when performing cfDNA size profiling using the dsDNA 930 Reagent kit on the Agilent Fragment Analyzer system, which allows higher resolution separation of DNA fragments in the range of 75 and 20,000 base pairs. The peaks at 35 and 10,000 bp correspond to the two internal size markers. The relative fluorescence (y-axis) of these markers is used to calculate the size of the unknown cfDNA samples (x-axis). Additional details of the cfDNA size analyses are summarized in Table 3.

**Figure 6:**
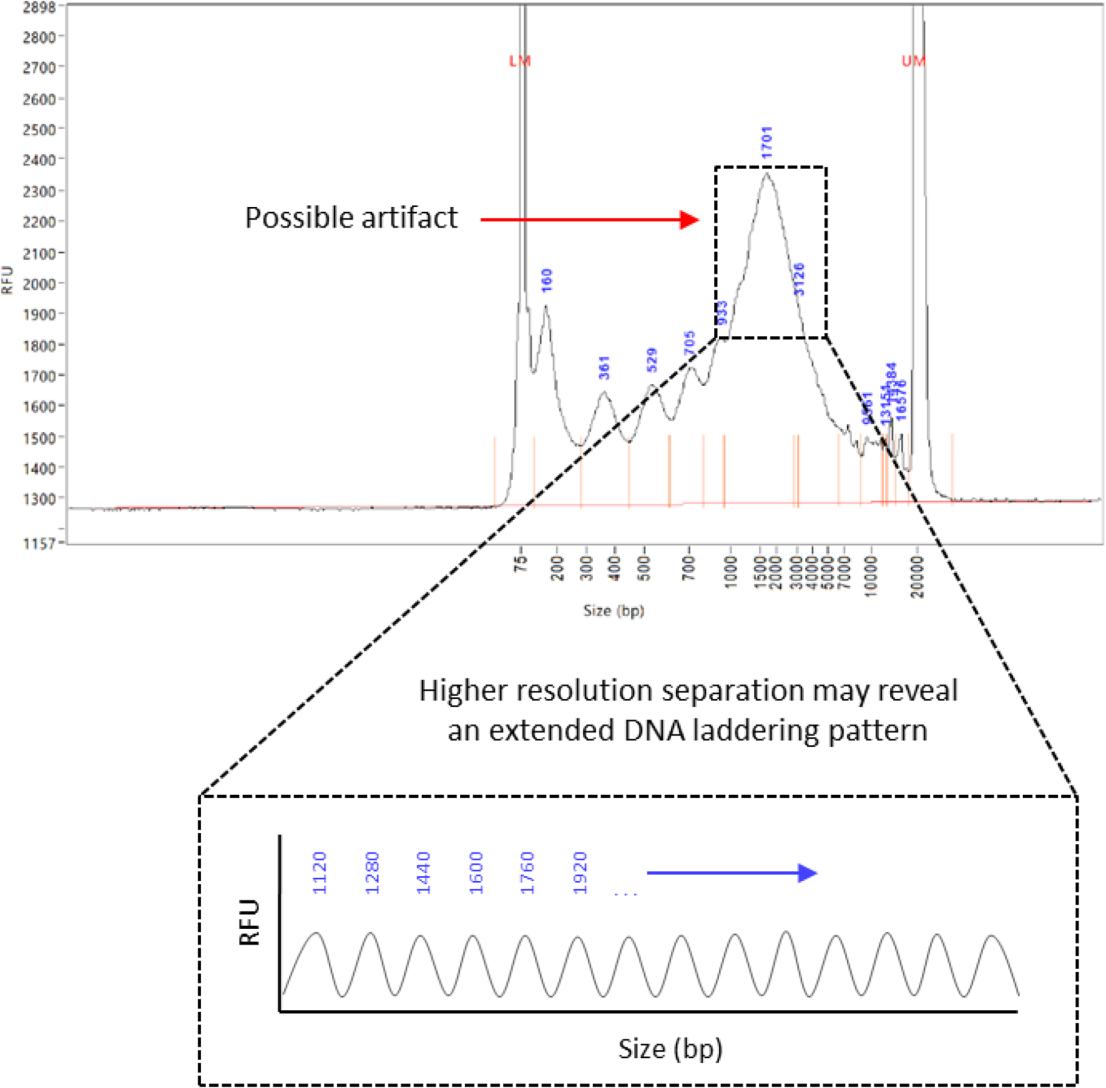
A cell-free DNA (cfDNA) peak that ranges between 1–6 kbp may be an artifact of incomplete size separation. Due to the resolution limitations of electrophoretic methods, several individual cfDNA populations are grouped into a single peak. Deconvolution of this peak through complete size separation may reveal an underlying DNA fragmentation profile that continues the series of DNA laddering.

**Figure 7:**
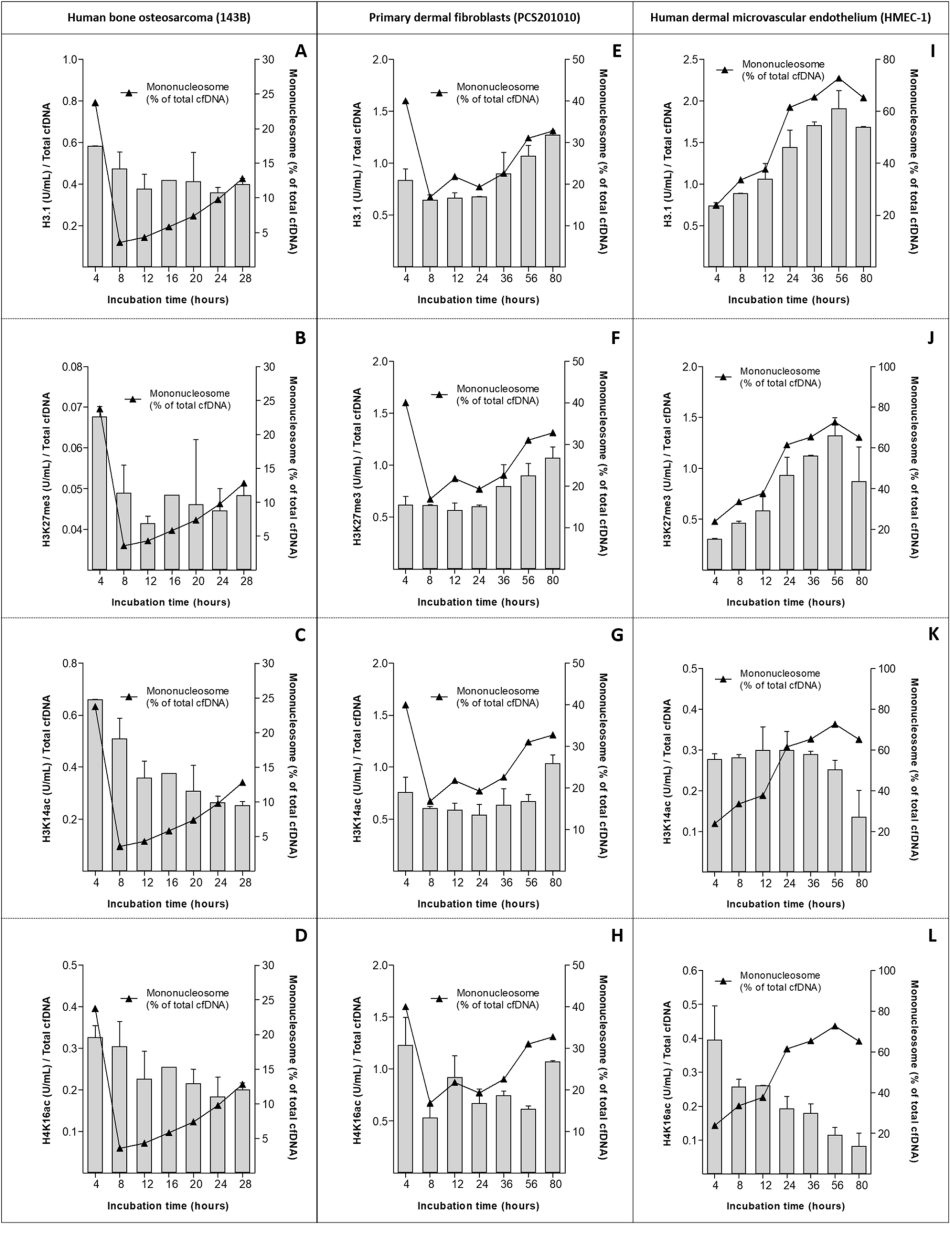
Levels of epigenetic marks normalized to total cfDNA. Serial measurements of cell-free nucleosomal histone variant H3.1 and PTMs (from top to bottom, H3.1, H3K27me3, H3K14ac, and H4K16ac) in the cell culture supernatants of **(A-D)** human bone osteosarcoma (143B) cells, **(E–H)** primary dermal fibroblasts (PCS201010), and **(I-L)** human dermal microvascular endothelial cells (HMEC-1). These epigenetic marks were measured directly from cell culture supernatant using Nu.Q Immunoassays (Belgian Volition SRL, Namur, Belgium). For all experiments, the values are expressed as total units/mL and are normalized to the total cfDNA levels, as determined by qPCR, at each corresponding time-point (left y-axis). The proportion of mono-nucleosomes (i.e., 50–250 bp) that comprise the total cfDNA population at each time-point for each cell line was calculated using the size gating function of the Agilent Bioanalyzer 2100 expert software and is plotted on the right y-axis. Error bars indicate standard deviation.

Taken together, these results indicate that mono-nucleosomes (~ 168 bp), di-nucleosomes (~ 343 bp), and tri-nucleosomes (~ 533 bp) originate mainly from apoptosis, while larger cfDNA fragments (~ 2858 bp) originate from necrosis. This is further substantiated by differences in the proportion of apoptotic vs necrotic cells and short vs. long cfDNA fragments. There were generally more apoptotic cells than necrotic cells, and this proportion increased over longer incubation periods (Table 4). Similarly, there were much higher levels of shorter than long fragments, and this proportion increased over time (Table 4).

**Table 4:** The relationship between the total number of necrotic vs apoptotic cells over increasing incubation periods in three cell lines, as determined by a caspase-3/7 assay, and the relationship between the concentration of 650–10,000 bp cfDNA fragments vs 50–250 bp cfDNA fragments at corresponding time-points, as determined by a capillary electrophoresis assay.

Cell line	Incubation time	Total necrotic cells/total apoptotic cells	cfDNA [650–10,000 bp] / [50–250 bp]
Human bone osteosarcoma (143B) cells	4 h	1.11	1.20
	8 h	0.87	11.12
	12 h	1.11	9.01
	16 h	1.54	7.54
	20 h	1.18	4.89
	24 h	1.82	3.19
	28 h	0.79	2.11
Primary dermal fibroblasts (PCS201010)	4 h	0.24	0.51
	8 h	0.30	2.07
	12 h	0.41	1.34
	24 h	0.14	1.30
	36 h	0.42	0.79
	56 h	0.45	0.50
	80 h	0.28	0.50
Human dermal microvascular endothelial cells (HMEC-1)	4 h	0.98	1.06
	8 h	1.82	0.60
	12 h	0.89	0.50
	24 h	0.75	0.13
	36 h	0.39	0.11
	56 h	0.39	0.07
	80 h	0.30	0.13

### Primary dermal fibroblasts (PCS201010)

PCS201010 cells showed increasing levels of total cfDNA, which correlated with increasing numbers of apoptotic and necrotic cells (Fig. 3E–H), and also exhibited the presence of four cfDNA size populations at all incubation periods (Fig. 5B and Table 3). This indicated the involvement of both apoptosis and necrosis in the generation of cfDNA. Similar to HMEC-1 cells, PCS201010 cells exhibited a higher ratio of apoptotic vs necrotic cells, which was also matched by an overall higher ratio of shorter to longer fragments (Tables 3 and 4). Furthermore, total cfDNA, as measured by Qubit but not by qPCR, showed a greater correlation with changes in the number of apoptotic cells (r = 0.92) than necrotic cells (r = 0.7530) (Supplementary Table S2). Unlike HMEC-1 cells, changes in total cfDNA were not as clearly reflected by changes in the concentration of differently sized cfDNA populations (Supplementary Table S3). However, changes in the levels of the combined concentration of cfDNA fragments ranging between 50–650 bp correlated significantly with changes in total cfDNA (r = 0.79), while levels of 650–10,000 bp cfDNA fragments did not correlate with total cfDNA (Supplementary Table S3). This indicated that apoptosis contributes only slightly more than necrosis toward the total cfDNA population. Furthermore, changes in 50–250 bp (r = 0.82), 250–450 bp (r = 0.7) and 50–650 bp (r = 0.78) cfDNA fragments correlated with changes in the number of apoptotic cells, while changes in none of the cfDNA populations correlated with changes in the number of necrotic cells (Supplementary Table S4). The reason why long fragments did not show a direct positive relationship with changes in necrotic cells may be as follows: After 24 h of incubation, there was a significant reduction in the number of necrotic cells (Fig. 3F,H), the same point at which 650–10,000 bp cfDNA fragments reached its highest levels (Table 3). Similarly, significant decreases in the number of necrotic cells between 56–80 h of incubation coincided with a significant increase in the level of 650–10,000 bp cfDNA fragments (Table 3). This suggests that the disintegration of necrotic cells, which results in lower levels of necrotic cells measured by the caspase-3/7 assay, is followed by the release of long cfDNA fragments into the cell culture medium.

Another reason for a lack of clear correlation between changes in the concentration of differently sized cfDNA populations and changes in apoptotic and necrotic cells may be that, despite higher cell numbers, more doublings and higher apoptosis and necrosis levels, PCS201010 cells exhibited very low cfDNA levels in comparison with HMEC-1 and 143B cells. The reason for this is unclear, but some hypotheses may be considered: (a) During apoptosis, the cytoplasm shrinks but the integrity of the organelles and plasma membrane remains preserved for a long period. Eventually, cellular constituents are divided and packaged into several spherical vesicles known as apoptotic bodies (ABs). In vivo, these ABs are typically engulfed and degraded by phagocytic cells. In in vitro conditions where there is an absence of macrophages, ABs usually enter a degenerative phase during which structural integrity is progressively lost, resulting in ballooning, permeabilization of the plasma membrane and eventually release of its cytoplasmic contents. This process is also known as secondary necrosis^43^. Variations in the duration of this process among different cells and conditions is well known. Thus, concerning apoptosis-derived cfDNA, it is possible that fibroblast-derived ABs maintain their integrity for a longer time-period in comparison to 143B and HMEC-1 cells. If this is the case, this capacity of fibroblasts may be facilitated by a mechanism such as the overexpression of transglutaminase which would increase cross-linking of cytoplasmic proteins and prolonged stability of ABs^44^. Thus, cfDNA levels in fibroblasts may be lower because the bulk of cfDNA is contained in ABs that may be missed by the cfDNA extraction procedure; (b) studies have shown that cfDNA can attach to the plasma membranes of various cell types^45^, and it is possible that the degree of cfDNA attachment may be more pronounced for fibroblast cells than 143B and HMEC-1 cells. Therefore, a large portion of cfDNA may remain bound to the plasma membrane when the cell culture supernatant is collected; (c) after release from dead/apoptotic cells, ABs or cfDNA may be taken up by live cells. Studies have shown that both cfDNA and ABs can be taken up by cells^31,46–49^; (d) higher nuclease activity in cell culture medium, resulting in rapid digestion of cfDNA as it enters the extracellular space; (e) higher nuclease activity intracellularly; and lastly (f) apoptosis and necrosis may occur without degradation of nuclear DNA^50,51^.

### Human bone osteosarcoma (143B) cells

In 143B cells, changes in the levels of total cfDNA, as measured by qPCR, correlated with changes in the levels of 250–450 bp (r = 0.89) and 450–650 bp (r = 0.92) cfDNA fragments but did not correlate with changes in the levels of 50–250 bp (r = 0.25) or 650–10,000 bp (r = 0.14) cfDNA fragments (Supplementary Table S3). The reason why changes in the levels of 50–250 bp cfDNA fragments showed no correlation with changes in total cfDNA levels is due to initially high levels after 4 h of incubation (Table 3). As will be explained later the initially high levels of 50–250 bp cfDNA fragments after 4 h of incubation and the significant reduction thereof after another 4 h of incubation may be the result of mitochondrial DNA digestion. Thus, between 8-28 h of incubation changes in the level of 50–250 bp cfDNA fragments correlated significantly with changes in total cfDNA levels (r = 0.91). The reason why 650–10,000 bp cfDNA fragments did not correlate with total cfDNA levels is because this population reached maximum levels after 16 h of incubation and declined thereafter (Table 3). Matching results were obtained for total cfDNA measured by the Qubit assay in all cases (Supplementary Table S3).

In contrast to HMEC-1 and PCS201010 cells, changes in total cfDNA levels in 143B cells did not correlate with changes in total apoptotic or necrotic cells when compared across the entire incubation period (Supplementary Table S2). Similarly, changes in the levels of differently sized cfDNA populations did not correlate with changes in the levels of apoptotic and necrotic cells (Supplementary Table S4). The reason for this is that the levels of apoptosis, necrosis, and differently sized cfDNA populations change significantly at specific incubation times (Fig. 3A–D) and cannot be accurately modelled by linear regression. After 4 h of incubation, a relatively high number of 143B cells are already apoptotic (Fig. 3A,C) and necrotic (Fig. 3B,D). As the number of apoptotic and necrotic cells decline over time up until 12 h of incubation, the level of cfDNA concomitantly increases. This indicates that, as apoptotic and necrotic cells are disintegrated and not detected by the assay anymore, their nuclear contents are released into the cell culture supernatant, resulting in increased levels of cfDNA. The majority of cfDNA is thus generated in the first 16 h of incubation and is likely the result of both apoptotic and necrotic cells. As cells start to divide rapidly after 16 h of incubation, only a small amount of cfDNA was generated for the remainder of the incubation period. Further increases in the number of apoptotic and necrotic cells suggests that apoptosis and necrosis is also responsible for the generation of these cfDNA molecules. However, in comparison with PCS201010 and HMEC-1 cells, 143B cells generally showed a significantly higher ratio of total necrotic vs apoptotic cells over the entire time-course experiment (Table 4), and concomitantly showed a higher ratio of longer cfDNA fragments to shorter cfDNA fragments (Tables 3 and 4).

While both apoptosis and necrosis appear to contribute to the extracellular presence of cfDNA in all cell lines, these results indicate that necrosis contributes more to the generation of cfDNA in 143B cancer cells, whereas apoptosis contributes more to the generation of cfDNA in non-cancer PCS201010 and HMEC-1 cells (Fig. 4). These results are supported by a recently published study in which necrosis was identified as the predominant mechanism involved in the generation of cfDNA, while apoptosis was found to contribute little to the total cfDNA population in some tumor types^52^.

### Cell-free DNA size analyses

Size profiling of cfDNA in each of the cell lines showed the simultaneous presence of at least four distinct cfDNA size populations at all incubation periods, including mono-nucleosomes (~ 180 bp), di-nucleosomes (~ 360 bp), tri-nucleosomes (~ 540 bp), and one population of slightly higher molecular weight ranging between 1–6 kbp with a modal size of ~ 3 kbp (Fig. 5). Levels of mono-, di- and tri- nucleosomes were found to be consistent with an origin from apoptosis, which is consistent with the literature^1–3^. However, the general assumption among researchers is that the larger ~ 3 kbp cfDNA fragments are the byproducts of randomly lysed cells, thus representing accidental genomic DNA contamination^53–58^. Alternatively, we have previously advocated the hypothesis that this cfDNA population may originate from live cells through an active extrusion mechanism^32,34,36^ and many others have suggested the same^30,33,59^. The notion of active DNA release is not new (reviewed in^1,60^). Several early studies have demonstrated levels of cfDNA in cell culture supernatant that do not correlate with dead or dying cells^39,60–65^, which indicated the involvement of a regulatory mechanism that controls the release of DNA into the extracellular space. Interestingly, these purportedly actively released cfDNA fragments have been shown to range between 0–6000 bp and demonstrated the typical apoptotic DNA laddering profile, which indicated the existence of two separate mechanisms for generating the same cfDNA profile^61,62^. DNA fragments that range between 150–6000 bp have also been found in the cargo of some extracellular vesicles^66–68^. In contrast, a study on the composition of exosomes found that cfDNA fragments with a modal size of 6 kbp are not associated with exosomes, but are instead actively released from cells through an autophagy- and multivesicular-endosome-dependent pathway^59^. In line with this we recently suggested that genome instability and micronuclei formation may serve as a precursor to the extracellular presence of ~ 3 kbp cfDNA fragments^32,69^.

However, the observations made in the current study indicate that cfDNA does not originate from live cells, but instead from dead or dying cells. Therefore, to gain better insight into this ~ 3 kbp DNA size population, we performed more precise sizing of 143B cfDNA using the dsDNA 930 Reagent kit on the Fragment Analyzer system, which offers increased resolution over the Bioanalyzer DNA high sensitivity assay (Fig. 5 A-2). In addition to the mono-, di- and tri- nucleosomes that are typically identified by the Bioanalyzer HS DNA assay, this assay revealed the presence of tetra—and penta-nucleosomes. We further analyzed 143B cfDNA using a combination of high volume cfDNA extraction and an optimized agarose gel electrophoresis method, which revealed the additional presence of hexa- and hepta-nucleosomes (Supplementary Fig. S3). In support of this, assessment of differently sized cfDNA populations using the Agilent Femto Pulse system, which is more sensitive than the Fragment Analyzer DNF-930–33—DNA 75-20,000 bp kit, also revealed up to 7 nucleosomal multiples (see application note: “cfDNA Separated on the Agilent Femto Pulse System”). These observations suggest that the commonly observed ~ 3 kbp cfDNA fragments are neither a distinct nor overrepresented cfDNA subpopulation but may rather be an artifact of incomplete size separation due to the resolution limitations of electrophoretic methods. Since it is difficult for these methods to separate the peaks corresponding to fragments longer than 7 nucleosomes, these ~ 3 kbp cfDNA fragment peaks may rather represent the sum of several overlapping individual oligo-nucleosomal peaks. This would suggest that the DNA laddering pattern seen up until 7 nucleosomes is continued for even longer fragments, generating a pattern highly similar to those generated by apoptosis. Such a hypothetical size profile is shown in Fig. 6.

As demonstrated earlier, these longer ~ 3 kbp cfDNA fragments appear to originate from necrotic cells. Although necrosis is generally characterized by non-specific cleavage of chromatin into large fragments, resulting in the appearance of a shearing pattern, several studies have demonstrated inter-nucleosomal cleavage of DNA in necrotic cells^70–73^. This raises the question of whether cleavage of these longer fragments may contribute to the shorter cfDNA pool that is generated by apoptosis. We did not assess cfDNA degradation kinetics in this study. However, levels of these longer cfDNA fragments declined at one point in all cell lines (Table 3), suggesting that they are degraded. Furthermore, despite varying levels of apoptosis and necrosis and changes in the proportion of differently sized cfDNA populations, all cell lines demonstrated a trend of decrease in the ratio of longer to shorter fragments over time (Fig. 5 and Table 4). Bearing in mind the DNA laddering pattern revealed by high-resolution size analysis (Fig. 5 A(2) and Supplementary Fig. S3), some of these longer fragments are likely degraded into several oligo-nucleosomal subunits, also including mono-, di-, and tri-nucleosomes. This was also observed in a recent study^52^.

Interestingly, 143B and PCS201010 cells demonstrated high levels of short sub-nucleosomal cfDNA fragments after 4 h of incubation which significantly declined after 8 h of incubation and showed even shorter modal sizes (Table 3), which explains the initially small ratio of long to short fragments as shown in Fig. 4. The reason for this decrease in size and concentration was not investigated in this study but one possible explanation is that it may represent digested mitochondrial DNA, although a portion of the size population may consist of mono-nucleosomes. In unpublished results, sequence analysis of cfDNA collected from 143B cells after 4 h of incubation showed a high coverage of the entire mitochondrial genome. In contrast, sequence analysis of cfDNA isolated after 24 h of incubation revealed no mitochondrial DNA sequences.

### Correlation between cfDNA and nucleosome histone variant H3.1 and PTM levels

As shown earlier (Table 1 and Fig. 2), cfDNA and nucleosome H3.1/PTM levels exhibit highly similar dynamics over increasing incubation periods suggesting they are one complex. However, notwithstanding similar trends, both cfDNA and many of the different nucleosome structures show varying levels at each time-point and different stepwise dynamics over increasing incubation times. This suggested that finer compositional changes in cfDNA and nucleosome structure over time may be elucidated by calculating changes in the ratio of cfDNA levels vs the levels of H3.1 and each of the PTMs at each of the time-points. This was done by dividing the total levels of H3.1, H3K27me3, H3K14ac, H4K16ac, respectively, with the corresponding levels of total cfDNA at each time-point. These results are summarized in Fig. 7.

In 143B cells, the ratio of H3.1 and each of the PTMs to cfDNA is initially high and decreases significantly in the period between 4–12 h of incubation, after which a relatively constant ratio is maintained until the end of the incubation period (Fig. 7A–D). Correlation of this data with cfDNA size analysis (Table 3) indicates that the initially high levels of each of these modifications correspond with an initially high ratio of mono-nucleosomes to larger cfDNA fragments. Incremental reductions in the ratio of these modifications to total cfDNA levels, despite an increase in larger-sized cfDNA fragments that reach peak levels after 16 h of incubation (Table 3), suggest that these modifications are richer in mono-nucleosomes, and poorer in larger fragments.

In PCS201010 cells, the ratio of H3.1 to total cfDNA is high after 4 h of incubation, declines slightly and maintains a constant ratio up until 24 h after which the ratio of H3.1 to cfDNA increases significantly and incrementally until the end of the time-course study. Unlike H3.1, the ratio of H3K27me3 to cfDNA is not higher after 4 h of incubation, but does also maintain a constant relationship from 8–24 of incubation, and then increases significantly and incrementally until the end of the time-course study. Changes in the ratios of H3K14ac and H4K16ac are slightly different. The ratio of H3K14ac to cfDNA is significantly increased after 4 h of incubation, decreases again slightly and maintains a constant ratio for the next 52 h of incubation (5 time-points), and then significantly increases again in the period between 56–80 h of incubation. The ratio of H4K16ac to cfDNA fluctuates over the entire incubation period, showing increased ratios after 4, 12, and 80 h of incubation (Fig. 7E–H). Similar to 143B cells, correlation with cfDNA size analysis data indicates that increases in the ratio of each of these modifications to total cfDNA generally correlates with increases in the ratio of mono-nucleosomes to larger cfDNA fragments.

In the case of HMEC-1 cells, the ratio of both H3.1 and H3K27me3 to cfDNA increases incrementally throughout the entire incubation period, but decreases between 56-80 h of incubation (Fig. 7I,J). A drop in the ratios between H3.1 and H3K27me3 to cfDNA corresponds with a drop in the ratio of mono-nucleosomes to larger fragments. In contrast, the ratio of H3K14ac to cfDNA remains constant over the entire incubation, period but also decreases between 56-80 h of incubation (Fig. 7K). This correlation suggests that H3K14ac is more abundant in mono-nucleosomes. However, a constant ratio of H3K14ac to total cfDNA, despite incremental increases in the ratio of mono-nucleosomes to larger cfDNA fragments up until 56 h of incubation suggests that cfDNA may become deacetylated at H3K14 over time. Lastly, the ratio of H4K16ac to cfDNA is initially high, but then decreases gradually over the course of the entire incubation period (Fig. 7L). Correlation with cfDNA size analysis data indicates that H4K16ac is not abundant in mono-nucleosomes.

These results seem to suggest that different cfDNA size populations possess different levels of epigenetic markers. One caveat surrounding these observations, however, is that the differently sized cfDNA populations were not separated and analyzed for H3.1 and PTMs independently in this study. Therefore, it cannot be stated with complete confidence whether differently sized cfDNA populations truly possess different levels of epigenetic markers, or whether larger fragments, for example, may exist in a structural conformation that sterically hinders access to antibodies, thereby resulting in biased measurements.

Three additional differences were observed between cell lines: (i) cfDNA from 143B cells contained significantly lower levels of H3K27me3 compared to PCS201010 and HMEC-1 cells; (ii) cfDNA from HMEC-1 cells contained significantly lower levels of H3K14ac compared to both 143B and PCS201010 cells; (iii) cfDNA from PCS201010 cells contained significantly higher levels of H4K16ac compared to both 143B and HMEC-1 cells. The reasons for these differences were not investigated this study.

## Summary

In this study we performed a serial analysis of cfDNA in three cell lines. Barring H3K14ac in 143B cells, total cfDNA levels correlated strongly with changes in H3.1 and each of the measured PTMs in all cell lines. This indicated that the majority of cfDNA fragments are not “naked” but exist in nucleosome structures that harbor the H3.1 variant as well as the H3K27me3, H3K14ac, and H4K16ac modifications. Temporal changes in the levels of these cfDNA-nucleosome complexes in each of the cell lines did not correlate with cell growth rate and does not appear to be constitutively released from live cells. Instead, changes in cfDNA levels over increasing incubation periods correlated with intermittent active and passive cell death events. Interestingly, while both apoptosis and necrosis were involved, apoptosis contributed more to the generation of cfDNA in non-cancer cell lines, while necrosis contributed more in the 143B cancer cell line. These observations were supported by matching changes in cfDNA size profiles. Four distinct cfDNA size populations were consistently observed in all cell lines, including mono-, di-, and tri-nucleosomes, and one larger population between 1–6 kbp with a modal size of ~ 3 kbp. Levels of mono-, di- and tri-nucleosomes were found to be governed mainly by apoptosis, whereas the levels of ~ 3 kbp cfDNA fragments were governed by necrosis. High-resolution size analysis of this larger cfDNA population revealed an underlying DNA laddering pattern extending up to seven nucleosomes in length, which likely extends further given the inherent resolution limitations of electrophoretic methods. This suggests that these commonly observed ~ 3 kbp cfDNA peaks are merely an artifact of incomplete size separation and is in fact composed of several oligo-nucleosome subpopulations. As this pattern is highly similar to those generated by apoptosis, it indicates that these oligo-nucleosomes likely contribute to the generation of mono-, di-, and tri-nucleosomes.

These observations may have some important implications. First, it challenges the general assumption that apoptosis is the main contributor towards the pool of circulating tumor DNA (ctDNA) in cancer patients. The notion that necrosis may contribute significantly to the ctDNA pool is consistent with at least three characteristics of tumor biology: (i) evasion of apoptosis is a hallmark of cancer cells, (ii) necrosis has proinflammatory and tumor-promoting potential^74^, and (iii) apoptotic cells and its contents are rapidly degraded by macrophages in vivo, whereas tumor necrosis leads to the release of cellular contents into the extracellular space^75,76^. Second, it challenges the general assumption that these larger cfDNA fragments are either (a) the byproducts of randomly lysed cells and thus genomic DNA contamination in cfDNA samples, or (b) the result of active secretion from live cells. In line with these points, it is noteworthy that this ~ 3 kbp cfDNA population, which is typically identified through size analysis using the Bioanalyzer HS DNA assay, is not only seen in cell cultures but is often encountered in various types of clinical biospecimens^53,54,56–58,77–79^. If the concentration of oligo-nucleosomes is too high in the latter case, samples are either discarded or several preanalytical approaches are followed to eliminate the longer fragments. Notwithstanding real contributions by accidental cell lysis, if it is true that necrosis results in the generation of oligo-nucleosomal cfDNA fragments, and that these fragments are not always degraded into mono-nucleosomes, necrotic cfDNA in the form oligo-nucleosomes that are derived from cancer cells may be excluded from analysis in clinical samples, resulting in the loss of important pathological information. On the other hand, if necrosis often results in the cleavage of DNA into mono-nucleosomes, or if longer necrosis-derived cfDNA fragments are digested into nucleosomes, necrotic cfDNA derived from non-target tissues or cells may significantly dilute the short-fragment ctDNA pool that is interrogated for mutational profiles in clinical samples, thereby reducing assay sensitivity and specificity.

In conclusion, these results support the notion that further enquiry into the release kinetics and physico-chemical properties of differently sized cfDNA populations in different contexts may advance our understanding of the biology of cfDNA and inform the development of increasingly powerful and comprehensive clinical assays.

## Limitations of this study and directions for future research

During this study we identified several factors that complicate kinetic assessments of cfDNA, thereby limiting exact determination of the relative contribution of apoptotic, necrotic and live cells toward the total cfDNA pool:

(1) cfDNA release was studied under normal conditions, wherein natural occurrences of apoptosis and necrosis are relatively low in comparison with artificially induced cell death. Low levels of both cell death and cfDNA levels may cause overlapping values, obfuscating subtle differences between processes. In these conditions, cells are also not highly synchronized in their cell cycles, which likely contributes to sporadic apoptosis and necrosis events and consequently irregular and somewhat unpredictable changes in cfDNA levels between different incubation periods; (2) There is a known delay in the release of cfDNA following the initiation of both apoptosis and necrosis, and these dynamic processes may not have been captured by the incubation periods selected in this study. Shorter incubation time intervals and/or longer total incubation periods for each cell line may be investigated to ensure that important dynamic changes are not missed. This also demonstrates the limitations of regression analysis for this type of data; (3) In line with the previous point, apoptotic bodies and extracellular vesicles were not characterized in this study, and it is not known what fraction of cfDNA is associated with these vesicles and how much of this population was lost during sample processing or cfDNA isolation. Parallel characterization of these vesicles and cfDNA would be ideal. Furthermore, commercial kits do not completely purify DNA from proteins and treatment of cell culture supernatant with proteinase K and sodium dodecyl sulfate (SDS) prior to extraction has been shown to significantly increase cfDNA yield, indicating an association with a protein complex^36,80^. Therefore, the level of cfDNA association with other protein complexes should also be assessed; (4) Necrotic DNA may be digested into DNA fragments that resemble apoptotic DNA; (5) Extracellular DNA degradation and digestion activities may fluctuate over time (e.g., endonucleases can be released by dead or dying cells, and activity may increase in response to an increase in cfDNA levels^81^); (6) cfDNA may be taken up by cells or adhere to cell surfaces, and there may differences in the behavior of apoptotic and necrotic DNA in this respect; (7) Various processes can inhibit the release of DNA, while other processes can speed up the degradation of DNA; (8) In addition to apoptosis and necrosis, various other forms of cellular demise can contribute to cfDNA release; (9) Different types of cell death result in the presence of differently sized cfDNA populations. As different preanalytical steps differently affect different cfDNA size populations (e.g., size bias of extraction kits^29,82^ and mechanical vulnerability of long fragments during processing^83^), this can potentially affect cfDNA measurements^29^; (10) It is difficult to determine the amount of apoptotic and necrotic cells that have completely disintegrated and/or newly emerged between incubation periods; (11) Apoptosis and necrosis can occur in the same cell^76^.

Apart from the fact that these variables are difficult to identify, monitor, and control, a recent paper has clearly shown how the complex interplay between all of these variables complicate the accurate assessment of cfDNA kinetics^52^. As such, assessment of all these variables was well beyond the scope of this work. There are thus still many open research questions and several blind spots in our knowledge of cfDNA biology that remain to be filled.

## Supplementary Information

Supplementary Information
