# Supplementary material for: Serial profiling of cell-free DNA and nucleosome histone modifications in cell cultures
Source: Sci Rep. 2021 May 4;11:9460. doi: 10.1038/s41598-021-88866-5 (PMC8096822; doi:10.1038/s41598-021-88866-5)
Supplement: Supplementary file 1 — Supplementary Information [file 41598_2021_88866_MOESM1_ESM.pdf]

Serial profiling of cell-free DNA and nucleosome histone modifications in cell cultures

Vida Ungerer [1,☯], Abel J. Bronkhorst [1,☯], Priscilla Van den Ackerveken [2], Marielle Herzog [2], Stefan Holdenrieder [*,1]

[1] Institute for Laboratory Medicine, German Heart Centre, Technical University Munich, Lazarettstraße 36, 80636 Munich, Germany

[2] Belgian Volition SPRL, 22 Rue Phocas Lejeune, Parc Scientifique Crealys, 5032 Isnes, Belgium

* Corresponding author email: holdenrieder@dhm.mhn.de

☯ Authors contributed equally

qPCR cfDNA measurements

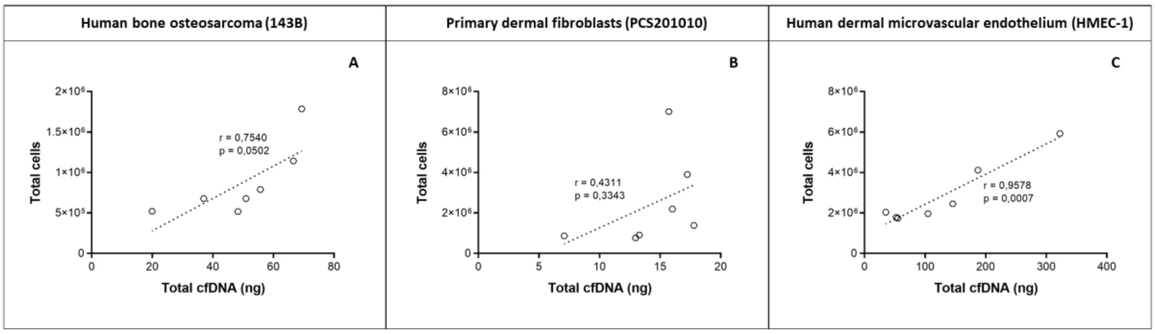

Qubit cfDNA measurements

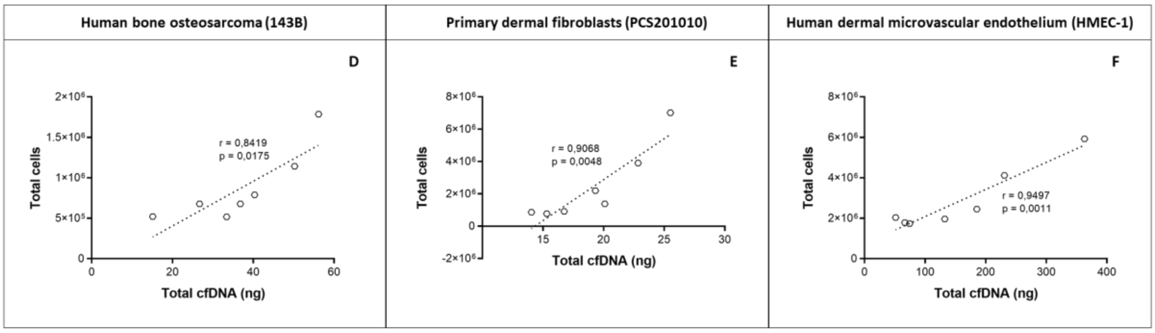

Supplementary Figure S1. Correlation between total cell-free DNA levels and total cell number. For each of the cell lines investigated in this study, including **(A, D)** human bone osteosarcoma (143B) cells, **(B, E)** primary dermal fibroblasts (PCS201010), and **(C, F)** human dermal microvascular endothelial cells (HMEC-1), total cfDNA levels (as determined by a **(A-C)** β-globin qPCR assay or **(D-F)** a Qubit dsDNA HS assay) present in cell culture supernatant collected at specific time-points were divided by the total number of cells present in the corresponding culture flask (as determined with the Guava Muse Cell Analyzer and the Cell Count and Viability assay). R-values close to 1 indicate perfect correlation, while R values close to zero indicate no correlation. P-values < 0.05 indicate statistical significance.

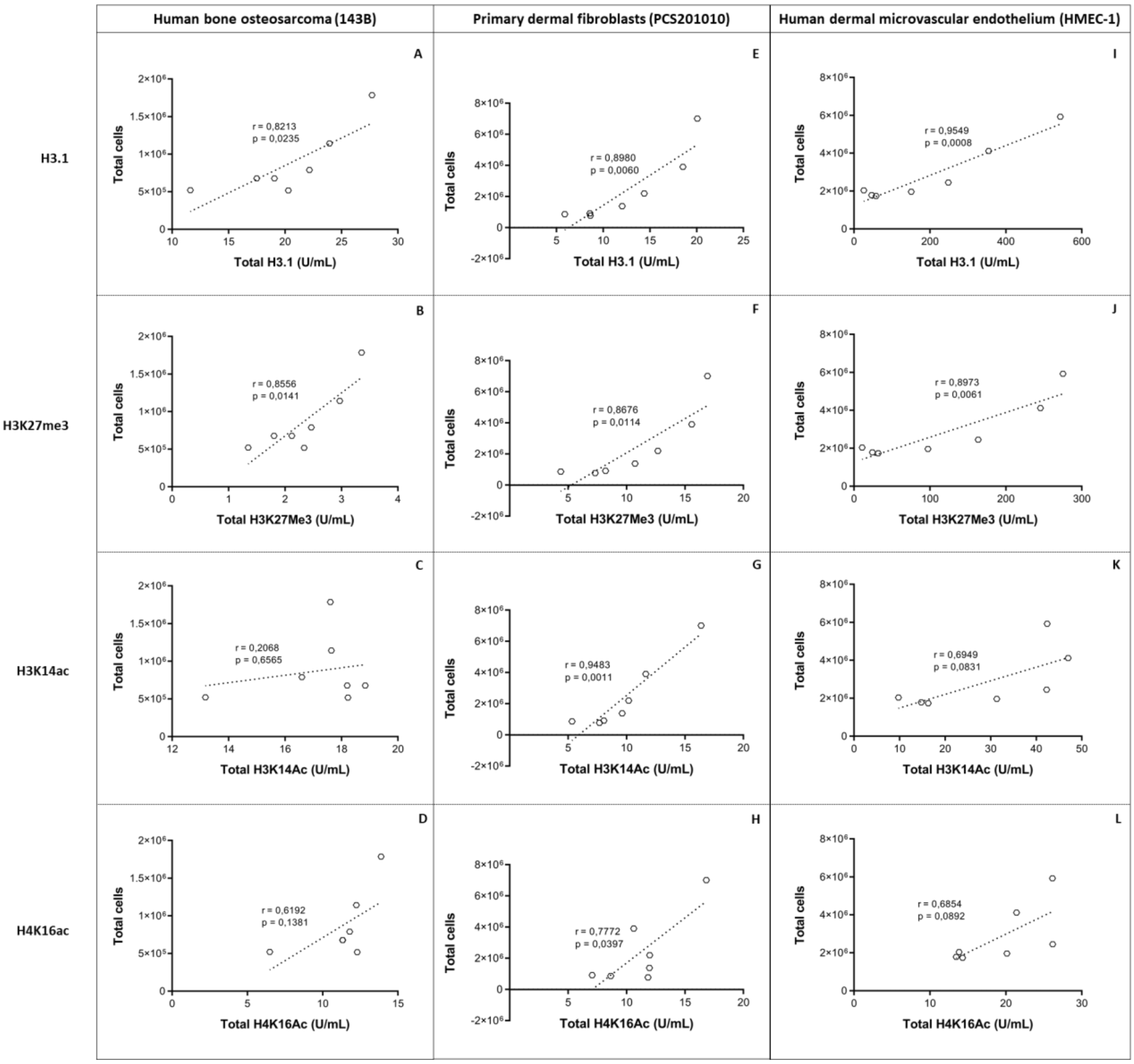

Supplementary Figure S2. Correlation between total H3.1 and histone PTM levels and total cells. Using chemiluminescence based Nu.Q Immunoassays, total levels of H3.1, H3K27me3, H3K14ac, and H4K16ac were measured directly from 50 µL of cell culture supernatant collected at different time points after increasing periods of incubation from **(A-D)** human bone osteosarcoma (143B) cells, **(E-H)** primary dermal fibroblasts (PCS201010), and **(I-L)** human dermal microvascular endothelial cells (HMEC-1). For each cell line, the total levels of H3.1 and histone PTM levels are plotted against the corresponding total cell number (as determined with the Guava Muse Cell Analyzer and the Cell Count and Viability assay) at each time point. R-values close to 1 indicate perfect correlation, while values R values close to zero indicate no correlation. P-values < 0.05 indicate statistical significance.

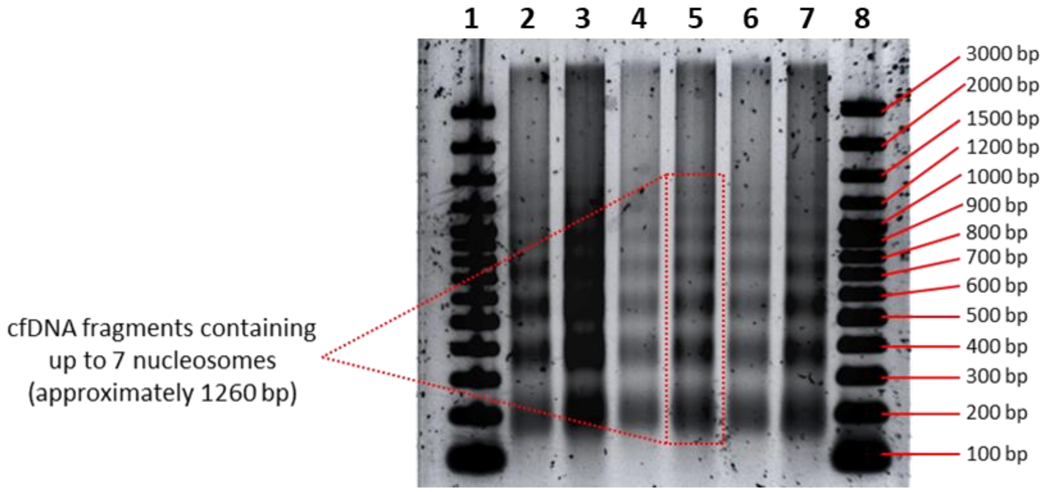

Supplementary Figure S3. Cell-free DNA separation and sizing by agarose gel electrophoresis. Cell-free DNA (cfDNA) was isolated from large volumes of human bone osteosarcoma (143B) cell culture supernatant and loaded onto an agarose gel (TBE, 1,5%) revealing a clear laddering pattern showing seven distinct cfDNA populations up to 7 nucleosomes in length. Lanes 1 and 8 contain the GeneRuler 100 bp Plus DNA ladder. Lanes 2, 4 and 6 were loaded with 60ng, 42ng, and 48ng cfDNA, respectively. Wells 3, 5 and 7 were loaded with larger volumes of the same cfDNA samples, amounting to 100ng, 70ng, and 80ng cfDNA, respectively.

Supplementary Table S1. Correlation between changes in the total number of apoptotic and necrotic cells over specific incubation periods in three cell lines, as determined by linear regression analysis.

Cell line	Time-frame	Correlation between total apoptotic vs total necrotic cells (Pearson's r)	p-value
Human bone osteosarcoma (143B)	4 to 28 h	0,6314	0,1283
	4 to 16 h	0,7323	0,2677
	16 to 28 h	0,5233	0,4767
Primary dermal fibroblasts (PCS201010)	4 to 80 h	0,8681	0,0113
	4 to 24 h	-0,6713	0,3287
	24 to 80 h	0,7906	0,2094
Human dermal microvascular endothelial cells (HMEC-1)	4 to 80 h	0,9081	0,0047
	4 to 36 h	-0,9255	0,0241
	36 to 80 h	0,9797	0,1284

Supplementary Table S2. Correlations between changes in total cfDNA levels, as determined by both qPCR and Qubit assay, and changes in the total number of apoptotic and necrotic cells, as determined by a caspase-3/7 assay, in three cell lines over specific time periods.

Cell line	cfDNA quantitation assay	Time-period	Variables compared	Pearson's r	p-value
Human bone osteosarcoma (143B)	Real-time qPCR (β-globin)	4-28 h	Total cfDNA vs total apoptotic cells	0,3708	0,4128
			Total cfDNA vs total necrotic cells	0,5242	0,2272
	Qubit HS DNA assay	4-28 h	Total cfDNA vs total apoptotic cells	0,4941	0,2597
			Total cfDNA vs total necrotic cells	0,5887	0,1644
Primary dermal fibroblasts (PCS201010)	Real-time qPCR (β-globin)	4-80 h	Total cfDNA vs total apoptotic cells	0,4764	0,2798
			Total cfDNA vs total necrotic cells	0,4787	0,2772
	Qubit HS DNA assay	4-80 h	Total cfDNA vs total apoptotic cells	0,9157	0,0038
			Total cfDNA vs total necrotic cells	0,753	0,0507
Human dermal microvascular endothelial cells (HMEC-1)	Real-time qPCR (β-globin)	4-80 h	Total cfDNA vs total apoptotic cells	0,977	0,0002
			Total cfDNA vs total necrotic cells	0,839	0,0183
		4-56 h	Total cfDNA vs total apoptotic cells	0,9249	0,0082
			Total cfDNA vs total necrotic cells	0,3809	0,4563
	Qubit HS DNA assay	4-80 h	Total cfDNA vs total apoptotic cells	0,9685	0,0003
			Total cfDNA vs total necrotic cells	0,8126	0,0263
		4-56 h	Total cfDNA vs total apoptotic cells	0,9255	0,0081
			Total cfDNA vs total necrotic cells	0,3598	0,4837

Supplementary Table S3. Correlation between changes in total cfDNA levels, as determined by both qPCR and Qubit assay, and changes in the concentration of differently sized cfDNA populations, as determined by a Bioanalyzer HS DNA assay, in three cell lines over specific time periods.

Cell line	cfDNA quantitation assay	Time-period	Variables compared	Pearson's r	p-value
Human bone osteosarcoma (143B)	Real-time qPCR (β-globin)	4-28 h	Total cfDNA vs [50-250 bp cfDNA]	0,2546	0,5817
			Total cfDNA vs [250-450 bp cfDNA]	0,892	0,0069
			Total cfDNA vs [450-650 bp cfDNA]	0,9206	0,0033
			Total cfDNA vs [50-650 bp cfDNA]	0,8269	0,0218
			Total cfDNA vs [650-10,000 bp cfDNA]	0,1042	0,824
		8-28 h	Total cfDNA vs [50-250 bp cfDNA]	0,9124	0,0112
	Qubit HS DNA assay	4-28 h	Total cfDNA vs [50-250 bp cfDNA]	0,3378	0,4587
			Total cfDNA vs [250-450 bp cfDNA]	0,9258	0,0028
			Total cfDNA vs [450-650 bp cfDNA]	0,9118	0,0042
			Total cfDNA vs [50-650 bp cfDNA]	0,8661	0,0117
			Total cfDNA vs [650-10,000 bp cfDNA]	0,04388	0,9256
		8-28 h	Total cfDNA vs [50-250 bp cfDNA]	0,9008	0,0143
Primary dermal fibroblasts (PCS201010	Real-time qPCR (β-globin)	4-80 h	Total cfDNA vs [50-250 bp cfDNA]	0,1558	0,7388
			Total cfDNA vs [250-450 bp cfDNA]	0,6987	0,0807
			Total cfDNA vs [450-650 bp cfDNA]	0,77	0,0429
			Total cfDNA vs [50-650 bp cfDNA]	0,5427	0,2081
			Total cfDNA vs [650-10,000 bp cfDNA]	0,3477	0,4448
	Qubit HS DNA assay	4-80 h	Total cfDNA vs [50-250 bp cfDNA]	0,6883	0,0873
			Total cfDNA vs [250-450 bp cfDNA]	0,7738	0,0412
			Total cfDNA vs [450-650 bp cfDNA]	0,5487	0,2022
			Total cfDNA vs [50-650 bp cfDNA]	0,7921	0,0337
			Total cfDNA vs [650-10,000 bp cfDNA]	-0,01158	0,9803
Human dermal microvascular endothelial cells (HMEC-1)	Real-time qPCR (β-globin)	4-80 h	Total cfDNA vs [50-250 bp cfDNA]	0,9735	0,0002
			Total cfDNA vs [250-450 bp cfDNA]	0,9896	<0,0001
			Total cfDNA vs [450-650 bp cfDNA]	0,9641	0,0005
			Total cfDNA vs [650-10,000 bp cfDNA]	0,8838	0,0083
		4-56 h	Total cfDNA vs [50-250 bp cfDNA]	0,9963	<0,0001
			Total cfDNA vs [250-450 bp cfDNA]	0,9823	0,0005
			Total cfDNA vs [450-650 bp cfDNA]	0,9036	0,0135
			Total cfDNA vs [650-10,000 bp cfDNA]	0,5226	0,2875
	Qubit HS DNA assay	4-80 h	Total cfDNA vs [50-250 bp cfDNA]	0,9843	<0,0001
			Total cfDNA vs [250-450 bp cfDNA]	0,9927	<0,0001
			Total cfDNA vs [450-650 bp cfDNA]	0,9495	0,0011
			Total cfDNA vs [650-10,000 bp cfDNA]	0,8574	0,0136
		4-56 h	Total cfDNA vs [50-250 bp cfDNA]	0,9956	<0,0001
			Total cfDNA vs [250-450 bp cfDNA]	0,9819	0,0005
			Total cfDNA vs [450-650 bp cfDNA]	0,8995	0,0146
			Total cfDNA vs [650-10,000 bp cfDNA]	0,5199	0,2904

Supplementary Table S4. Correlation between changes in the concentration of differently sized cfDNA populations, as determined by a Bioanalyzer HS DNA assay, and changes in the total number of apoptotic and necrotic cells, as determined by a caspase-3/7 assay, in three cell lines over specific time periods.

Cell line	Time-period	Variables compared	Pearson's r	p-value
Human bone osteosarcoma (143B)	4-28 h	[50-250 bp cfDNA] vs total apoptotic cells	0,5573	0,1937
		[250-450 bp cfDNA] vs total apoptotic cells	0,4698	0,2874
		[450-650 bp cfDNA] vs total apoptotic cells	0,23	0,6199
		[650-10,000 bp cfDNA] vs total apoptotic cells	-0,4294	0,3363
		[50-250 bp cfDNA] vs total necrotic cells	0,7174	0,0695
		[250-450 bp cfDNA] vs total necrotic cells	0,6105	0,1454
		[450-650 bp cfDNA] vs total necrotic cells	0,4738	0,2828
		[650-10,000 bp cfDNA] vs total necrotic cells	-0,1295	0,782
Primary dermal fibroblasts (PCS201010)	4-80 h	[50-250 bp cfDNA] vs total apoptotic cells	0,822	0,0233
		[250-450 bp cfDNA] vs total apoptotic cells	0,7008	0,0794
		[450-650 bp cfDNA] vs total apoptotic cells	0,3522	0,4385
		[50-650 bp cfDNA] vs total apoptotic cells	0,7849	0,0365
		[650-10,000 bp cfDNA] vs total apoptotic cells	-0,2396	0,6049
		[50-250 bp cfDNA] vs total necrotic cells	0,5267	0,2246
		[250-450 bp cfDNA] vs total necrotic cells	0,5922	0,1613
		[450-650 bp cfDNA] vs total necrotic cells	0,2747	0,5511
		[50-650 bp cfDNA] vs total necrotic cells	0,5748	0,177
		[650-10,000 bp cfDNA] vs total necrotic cells	-0,4599	0,2991
Human dermal microvascular endothelial cells (HMEC-1)	4-80 h	[50-250 bp cfDNA] vs total apoptotic cells	0,934	0,0021
		[250-450 bp cfDNA] vs total apoptotic cells	0,9389	0,0017
		[450-650 bp cfDNA] vs total apoptotic cells	0,9481	0,0011
		[650-10,000 bp cfDNA] vs total apoptotic cells	0,9057	0,005
		[50-250 bp cfDNA] vs total necrotic cells	0,7614	0,0467
		[250-450 bp cfDNA] vs total necrotic cells	0,7686	0,0435
		[450-650 bp cfDNA] vs total necrotic cells	0,8519	0,015
		[650-10,000 bp cfDNA] vs total necrotic cells	0,8597	0,0131
	4-56 h	[50-250 bp cfDNA] vs total apoptotic cells	0,9504	0,0036
		[250-450 bp cfDNA] vs total apoptotic cells	0,8471	0,0333
		[450-650 bp cfDNA] vs total apoptotic cells	0,7295	0,0998
		[650-10,000 bp cfDNA] vs total apoptotic cells	0,406	0,4245
		[50-250 bp cfDNA] vs total necrotic cells	0,4304	0,3943
		[250-450 bp cfDNA] vs total necrotic cells	0,2581	0,6215
		[450-650 bp cfDNA] vs total necrotic cells	0,2037	0,6987
		[650-10,000 bp cfDNA] vs total necrotic cells	0,02114	0,9683
